# Interplay between the catabolite repression control protein Crc, Hfq and RNA in Hfq-dependent translational regulation in *Pseudomonas aeruginosa*

**DOI:** 10.1093/nar/gkx1245

**Published:** 2017-12-13

**Authors:** Elisabeth Sonnleitner, Alexander Wulf, Sébastien Campagne, Xue-Yuan Pei, Michael T Wolfinger, Giada Forlani, Konstantin Prindl, Laetitia Abdou, Armin Resch, Frederic H -T Allain, Ben F Luisi, Henning Urlaub, Udo Bläsi

**Affiliations:** 1 Department of Microbiology, Immunobiology and Genetics, Max F. Perutz Laboratories, University of Vienna, Vienna Biocenter, Dr. Bohrgasse 9, 1030 Vienna, Austria; 2 Biophysical Mass Spectrometry Group, Max Planck Institute for Biophysical Chemistry, 37077 Göttingen, Germany; 3 Institute of Molecular Biology and Biophysics, ETH Zürich, 8093 Zürich, Switzerland; 4 Department of Biochemistry, University of Cambridge, Cambridge CB2 1GA, UK; 5 Institute of Theoretical Chemistry, University of Vienna, 1090 Vienna, Austria; 6 Center for Anatomy and Cell Biology, Medical University of Vienna, 1090 Vienna, Austria; 7 Department of Fundamental Microbiology, University of Lausanne, 1015 Lausanne, Switzerland; 8 Bioanalytics, Institute for Clinical Chemistry, University Medical Center Göttingen, 37075 Göttingen, Germany

## Abstract

In *Pseudomonas aeruginosa* the RNA chaperone Hfq and the *c*atabolite *r*epression *c*ontrol protein (Crc) act as post-transcriptional regulators during carbon catabolite repression (CCR). In this regard Crc is required for full-fledged Hfq-mediated translational repression of catabolic genes. RNA_seq_ based transcriptome analyses revealed a significant overlap between the Crc and Hfq regulons, which in conjunction with genetic data supported a concerted action of both proteins. Biochemical and biophysical approaches further suggest that Crc and Hfq form an assembly in the presence of RNAs containing A-rich motifs, and that Crc interacts with both, Hfq and RNA. Through these interactions, Crc enhances the stability of Hfq/Crc/RNA complexes, which can explain its facilitating role in Hfq-mediated translational repression. Hence, these studies revealed for the first time insights into how an interacting protein can modulate Hfq function. Moreover, Crc is shown to interfere with binding of a regulatory RNA to Hfq, which bears implications for riboregulation. These results are discussed in terms of a working model, wherein Crc prioritizes the function of Hfq toward utilization of favored carbon sources.

## INTRODUCTION

The opportunistic pathogen *Pseudomonas aeruginosa* can utilize numerous carbon sources, which permits growth under diverse environmental conditions. The uptake and assimilation of carbon is controlled by carbon catabolite repression (CCR), a process that ensures that the utilization of less preferred carbon sources (e.g. mannitol or acetamide) is impeded until the preferred one (e.g. succinate) is consumed (1). CCR not only impacts on metabolic regulation, but is also linked to complex behavior including biofilm formation, quorum sensing, virulence and antibiotic susceptibility (2–5).

In contrast to CCR in Enterobacteriaceae and Firmicutes (6), in *Pseudomonas* CCR operates at the post-transcriptional level and employs the RNA chaperone Hfq, the catabolite repression control protein Crc and the regulatory RNA CrcZ (1,7). Recent studies provided evidence that Hfq acts as a translational repressor during CCR in *P. aeruginosa* (7). During growth on succinate several catabolic genes of *P. aeruginosa* were shown to be translationally silenced by Hfq, which can bind with its distal face to A-rich sequences within or adjacent to ribosome binding sites. Upon relief of CCR, e.g. after exhaustion of succinate and continued growth on mannitol, the level of the Hfq-binding RNA CrcZ increases (8), leading to sequestration of Hfq. This in turn abrogates Hfq-mediated translational repression of the respective catabolic genes (7).

The catabolite repression control protein Crc has been implicated in multicellular behavior and biofilm formation. A *crc* mutant was impaired in swimming, swarming and twitching motility, and showed defects in initial biofilm development (2,4,9). Some of these phenotypes were also observed with a PAO1*hfq-* mutant (10), indicating early on that Hfq and Crc may act together. In fact, recent studies suggested that both, Hfq and Crc, mediate post-transcriptional regulation during CCR as full-fledged repression of catabolic genes by Hfq required Crc (7,11).

In Enterobacteriaceae, Hfq is pivotal for riboregulation, which results on the one hand from binding to and protection of small regulatory RNAs (sRNA) from nucleolytic decay, and on the other hand from accelerating the annealing between sRNAs and their target mRNAs (reviewed in 12). The *P. aeruginosa* Hfq, which lacks the C-terminal extension present in enterobacteriaceael counterparts, has likewise been shown to accelerate annealing of two RNA substrates (13,14) as well as being required for riboregulation of *antR* mRNA by the sRNAs PrrF1–2 (14) and of *algC* mRNA by the sRNA ErsA (15). Interactome studies performed with *Escherichia coli* Hfq (16) and *P. aeruginosa* Hfq (17) revealed a large number of putative interacting proteins with functions in transcription, translation and mRNA decay. Several candidate proteins including RNA polymerase, ribosomal protein S1 (18), RNase E (19), polyA-polymerase and polynucleotide-phosphorylase (20) have been found to associate with *E. coli* Hfq. However, several follow up studies did not reveal a direct physical interaction between Hfq and these candidate proteins (21–23). Most likely, these complexes are RNA-mediated and result from the spatial association of the transcriptional, translational and RNA decay machineries. Similarly, in *P. aeruginosa* components of the degradosome were shown to co-purify with Hfq (17). Interestingly, the *P. aeruginosa* Crc protein co-purified as well with tagged Hfq protein (17). Moreover, pull-down assays indicated that *P. putida* Hfq and Crc form a co-complex in the presence of CrcZ RNA (11).

Here, using an *E. coli* two-hybrid system and co-immunoprecipitation (co-IP), we first show that Hfq and Crc associate *in vivo*. Biochemical and biophysical experiments extend findings from recent studies (11), and show that the Hfq/Crc interaction requires RNA bound to the distal side of Hfq. Crc bound neither to Hfq nor to RNA alone (24). However, as revealed by RNA cross-linking, Crc interacts with both Hfq and RNA in Hfq/Crc/RNA complexes. The multivalency inherent to Crc apparently increases the stability of these complexes when compared with Hfq/RNA complexes, whereas it appears not to affect selectivity. Moreover, our data indicate that Crc interferes with the binding of a sRNA to Hfq. The implications of the latter finding for riboregulation are discussed.

## MATERIALS AND METHODS

### Bacterial strains, plasmids and growth conditions

The strains and plasmids used in this study are listed in Supplementary Table S1. Details on the construction of plasmids and strains are provided in Supplementary Text S1. Unless indicated otherwise, the cultures were grown at 37°C in BSM medium (30.8 mM K_2_HPO_4_, 19.3 mM KH_2_PO_4_, 15 mM (NH_4_)_2_SO_4_, 1 mM MgCl_2_ and 2 μM FeSO_4_) supplemented with the indicated carbon sources. If required, *E. coli* was grown in the presence of 100 μg ml^−1^ ampicillin, 25 μg ml^−1^ tetracycline and 25 μg ml^−1^ kanamycin, respectively and *P. aeruginosa* was grown in the presence of 50 μg ml^−1^ gentamicin, 100 μg ml^−1^ tetracycline and 250 μg ml^−1^ carbenicillin, respectively.

### RNA_seq_ library construction and sequence analysis

Total RNA was prepared from two biological replicates of strains PAO1, PAO1Δ*crc* and PAO1*hfq*-, respectively, after growth in BSM complex medium (BSM medium containing 40 mM succinate, 5 mM of acetate, glucose, mannitol, acetamide, histidine, tryptophan, phenylalanine, leucine, isoleucine, glutamate, arginine, valine and lysine, 0.25 mM anthranilate and 0.25% glycerol) to an OD_600_ of 1.5. Then, 10 ml samples were withdrawn and total RNA was extracted using the hot phenol method (25), contaminating DNA was removed by DNase (Roche) treatment followed by phenol-chloroform (pH 5.5) extraction and ethanol precipitation. To remove ribosomal RNAs, the Ribo-Zero™ Magnetic Kit for Gram-negative bacteria (Epicentre) was used according to the manufacturer's instructions. Libraries were constructed using NEBNext^®^ Ultra™ Directional RNA Library Prep Kit from Illumina. 100 base pair single end sequence reads were generated using the Illumina HiSeq 2000 platform at the Vienna BioCenter Core Facility (http://www.csf.ac.at). Sequencing adapter removal was performed with cutadapt (26). Mapping of the samples against the PAO1 reference genome (NCBI accession number NC_002516.2) was performed with Segemehl (27) with default parameters. Reads mapping to regions annotated as either rRNA or tRNA were discarded from all data and ignored for all follow up analysis steps. The mapped sequencing data were prepared for visualization using the ViennaNGS tool box, and visualized with the UCSC Genome Browser (28). Reads per gene were counted using BEDTools (29) and the Refseq annotation of *P. aeruginosa* (NC_002516.2). Differential gene expression analysis was performed with DESeq (30). All RNAs with a fold-change greater than 5 and a multiple testing adjusted *P*-value below 0.05 were considered to be differentially abundant. The raw sequencing data were deposited in the European nucleotide archive (ENA) as a study under the accession number PRJEB22802.

### Bacterial adenylate cyclase-based two-hybrid system (BACTH)

Plasmids encoding C- and N-terminal fusion-proteins of Hfq and Crc with the catalytic domains T25 and T18, respectively, of *Bordetella pertussis* adenylate cyclase were constructed (Supplementary Text S1) and co-transformed into the *cya* deficient *E. coli* strain BTH101. BACTH was performed as previously described (31). Briefly, the interaction between two hybrid proteins was quantified by determining the β-galactosidase activity, which in turn depends on the intracellular cAMP levels. *Escherichia coli* strain BTH101, harboring the respective plasmids, was grown in Luria broth (32) to an OD_600_ of 0.7. Then, 1 mM IPTG was added, and 1 h thereafter the cells were harvested and the β-galactosidase activity was determined.

### β-galactosidase assays

The β-galactosidase activities were determined as described (32). The cells were permeabilized with 5% toluene. Unless indicated otherwise, the β-galactosidase units in the different experiments were derived from three independent experiments and are shown as mean. The error bars in the different Figures represent standard deviations.

### *In vivo* and *in vitro* co-immunoprecipitation studies

For *in vivo* co-IP, PAO1, PAO1Δ*hfq* and PAO1Δ*crc* were grown in BSM complex medium as described above (40 ml of culture) and harvested at an OD_600_ of 1.5. The cells were first washed in lysis buffer (20 mM Tris pH 8.0, 150 mM KCl, 1 mM MgCl_2_, 1 mM DTT, 0,05% Triton X-100) and then snap frozen in liquid nitrogen. The cells were lysed by sonication (six times for 10 s on ice) in 800 μl lysis buffer in the presence of 200 U RiboLock^®^ RNase inhibitor (Fermentas). Cell debris were removed by centrifugation and anti-Hfq antibodies (Pineda) were added to 60 μl supernatant and incubated for 2 h at 4°C on a rotating wheel. Then, 5 μl Dynabeads^®^ Protein G beads (Novex) were added and the incubation was continued for 1 h. The beads were washed three times with lysis buffer and finally collected in 25 μl of SDS loading dye. 5 μl were used for further analysis by western-blotting.

For *in vitro* co-IP studies, 40 pmol of Hfq-hexamer (Hfq_6_) and 120 pmol of Crc protein with or without 40 pmol RNA were incubated for 30 min at 37°C in 200 μl ES-buffer (10 mM Tris pH 8.0, 10 mM KCl, 40 mM NaCl and 1 mM MgCl_2_) in the presence of 0.05% Triton X-100. Then, 10 μl of rabbit anti-Hfq antibodies (Pineda) were added and the incubation was continued for 30 min at 4°C on a rotating wheel. Thereafter, 5 μl Dynabeads^®^ Protein G beads (Novex) were added for 30 min. The beads were washed three times with ES-buffer and finally collected in 50 μl of SDS loading dye. 5 μl were used for further analyses by western-blotting.

### Western-blot analyses

Equal amounts of co-immunoprecipitated or total proteins were separated on 12% SDS-polyacrylamide gels, and then electro-blotted onto a nitrocellulose membrane. The blots were blocked with 5% dry milk in TBS buffer, and probed with rabbit anti-Hfq (Pineda), or rabbit anti-Crc (Pineda) antibodies. Immunodetection of ribosomal protein S1 served as a loading control. The antibody-antigen complexes were visualized with alkaline-phosphatase conjugated secondary antibodies (Sigma) using the chromogenic substrates nitro blue tetrazolium chloride (NBT) and 5-Bromo-4-chloro-3-indolyl phosphate (BCIP).

### NMR

The Crc protein was produced in *Escherichia coli* BL21(DE3)(pET26bII-Crc). Crc was deuterated by growing the cells in M9 minimal medium containing 100% D_2_O (∼80% of the protons were replaced by deuterium), in the presence of ^15^N-labeled NH_4_Cl (1g/l) or in the presence of alpha-ketoisovaleric acid (^13^C_5_, 98%; 3-D_1_, 98%) to ensure specific ^13^C-labeling of leucine and valine methyl groups. The cells were grown at 37°C to an OD_600_ of 0.6. Then IPTG was added to a final concentration of 0.5 mM for 18 h at 15°C. The protein was then purified as described (33). All NMR measurements were performed at 313 K on an Avance III 900 MHz spectrometer. For the NMR titration of ^15^N-labeled Crc by Hfq, the ^15^N–^1^H fingerprint of Crc was monitored upon addition of Hfq by recording 2D ^15^N–^1^H BEST-TROSY HSQC. For the titration of ^13^C-labeled Crc, the signals for Crc were monitored upon addition of the Hfq/RNA complex by recording 2D ^13^C–^1^H HMQC spectra.

### Microscale thermophoresis (MST)

MST is based on the directed movement of molecules along temperature gradients. Any change of the hydration shell of biomolecules due to changes in their structure/conformation results in a relative change of the movement along the temperature gradient, which can be used to determine binding affinities (34). 20 μM of purified Crc and Hfq proteins were labeled with Monolith NT™ Protein Labeling Kit RED-NHS according to the manufacturer's instructions (Nano Temper). To study Hfq-sRNA interactions, PrrF2 sRNA was labeled at the 3′-end using T4 RNA Ligase (NEB) and pCp-Cy5 (Jena Bioscience) according to the manufacturer′s instructions. For determination of protein-protein or protein-RNA interactions 40 nM PrrF2-Cy5, 30 nM labeled Hfq and—due to a weaker labeling efficiency—200 nM labeled Crc protein, respectively, were used in the presence of increasing amounts of ligands (either Hfq, Crc, *amiE*_6ARN_ RNA-oligonucleotide (5′-AAAAAUAACAACAAGAGG-3′; purchased from Sigma) or combinations thereof, as indicated in the Results. The ligands were dissolved in ES-buffer (10 mM Tris pH 8.0, 10 mM KCl, 40 mM NaCl and 1 mM MgCl_2_) in the presence of 0.05% Tween-20. After 2 min incubation at room temperature, the samples were loaded onto MST Premium coated capillaries (Nano Temper) and measured in a MST Monolith NT.115 instrument at the Vienna BioCenter Core Facility (http://www.csf.ac.at). The MST measurements were performed in duplicate. If not indicated otherwise, the following parameters were used: LED Power 90%, MST Power 60%. Data analysis was performed with NTAffinityAnalysis v2.0.2 for thermophoresis and T-jump analysis 0 and 5 s after the pulse. For determination of the *K*_d_-values the Hill Model was used that is included in the NTAffinityAnalysis software.

### RNA-Protein cross-linking

To reconstitute the RNA-protein complex, 1 nmol Hfq and 1 nmol *amiE*_6ARN_ RNA were incubated with 3 nmol Crc in 200 μl ES-buffer for 30 min at 37°C. Substituting Crc with additional ES-buffer served as a negative control. Samples were split evenly into two aliquots, one of which was UV-irradiated at 254 nm, while the other served as a non-irradiated control. Further sample processing was performed as described in detail in Sharma *et al.* (35).

### Protein-Protein cross‐linking

Freshly prepared Hfq**/**Crc**/***amiE*_6ARN_ complexes were used for chemical cross‐linking with the amine‐reactive, water‐soluble, homobifunctional protein cross‐linker bis(sulfosuccinimidyl)suberate (BS3; Thermo Fisher Scientific). A 263-times molar excess of the cross‐linker over the Hfq**/**Crc**/***amiE*_6ARN_ complex was used; 480 pmol of the Hfq**/**Crc**/***amiE*_6ARN_ complex was incubated with 126 nmol of BS3 and incubated on ice for 2 h. After the incubation period, the reaction was quenched by addition of 1 μl of 200 mM Tris–HCl (pH 8.0). The cross‐linked samples were then subjected to SDS-PAGE on a 4–12% Bis–Tris gel (Invitrogen) with MOPS as running buffer. The gel was stained using Coomassie brilliant blue G250 and de-stained in water. The identified bands, corresponding to cross‐linked protein-protein conjugates, were excised and subjected to in‐gel trypsin or trypsin/Lys-C digestion in a 1:5 ratio of total protein to the enzyme mix (Trypsin LysC Mix; Promega). The proteolytic peptides were extracted and resuspended in 2% acetonitrile (ACN) and 0.05% trifluoro acetic acid (TFA) in a final volume of 14 μl and subjected to liquid chromatography-tandem mass spectrometry (LC–MS/MS) analysis.

### Mass spectrometry (MS/MS) and MS data analysis

The samples were loaded onto a self-packed C18 column, mounted on a Dionex Ultimate 300 UHPLC^+^ (Thermo Scientific): 3 μm pore size, 75 μm in diameter, 30 cm in length (Reprosil-Pur^®^ 120C18-AQ, Dr Maisch GmbH). The peptides were separated by reverse-phase chromatography on a 58 min multi-step gradient with a flow rate of 0.3–0.4 μl/min before entering the mass spectrometer (QExactive HF, Thermo Scientific). MS1 spectra were recorded in profile mode with a resolution of 120k, whereas MS2 spectra were recorded in centroid mode with a resolution of 30k. The isolation window was set to 1.6 *m/z* and the dynamic exclusion was set to 9 s. The raw data of RNA–protein heteroconjugates were analyzed and manually validated with the OpenMS pipeline RNPxl (36). The raw data of protein-protein crosslinked spectra were analysed and validated using pLink (37). Structural visualization of results was performed with Chimera 1.1.2. (38).

### Electrophoretic mobility shift assays (EMSA)

For *in vitro* transcription of CrcZ (426 nt) and PrrF2 (107 nt) RNA the AmpliScribe T7-Flash Transcription Kit (Epicentre Biotechnologies) was used according to the manufacturer′s instructions. First, PCR fragments were generated with the primer pairs E6 (5′-TCT AGA CGT AAT ACG ACT CAC TAT AGG CAC AAC AAC AAT AAC AAG C-3′) and C6 (5′-**ATG CGG ATC C**GA AAT GGT GTA AGG CGA AGG-3′) (*crcZ*) and W77 (5′-TTT TCT AGA CGT AAT ACG ACT CAC TAT AGG ACT GGT CGC GAG GCC-3′) and X77 (5′-CAA AAA AAG ACC CGG CAA AG-3) (*prrF2*) and chromosomal DNA of PAO1. The forward primers contain a T7 promoter sequence (underlined).

To determine whether Crc affects Hfq-RNA complex formation, the RNA oligonucleotide *amiE*_6ARN_ was 5′-end labeled with [γ-^32^P]-ATP (Hartmann Analytic) and polynucleotide kinase (Thermo Scientific), and the labeled RNA was extracted using phenol-chloroform followed by ethanol precipitation. 10 nM labeled RNA was incubated in ES buffer (10 mM Tris pH 8.0, 10 mM KCl, 40 mM NaCl and 1 mM MgCl_2_) with increasing amounts of purified Hfq protein as specified in the legend to Figure 4A in the presence or absence of 480 nM Crc (3-fold molar excess over the highest concentration of Hfq) and 25 ng tRNA in a total volume of 10 μl.

To assess the stability of RNA/Hfq and RNA/Hfq/Crc assemblies (Figure 4D), the complexes were pre-formed as described above using 80 nM Hfq_6_, 10 nM *amiE*_6ARN_ RNA in the presence or absence of 480 nM Crc in a 60 μl reaction volume containing 12 μl loading dye (see above). After 2 min pre-incubation, 10 μl were loaded on a 4% native polyacrylamide gel and then 100 nM unlabeled *amiE*_6ARN_ RNA was added. 10 μl samples were loaded 15, 45, and 120 sec thereafter. The gel was run continuously during the experiment.

For simultaneous detection of PrrF2 and *amiE*_6ARN_ RNA (Figure 6D), PrrF2 was labeled at the 3′-end with pCp-Cy5 as mentioned above. The *amiE*_6ARN_ RNA oligonucleotide labeled at the 5′-end with 6-carboxyfluorescein (6-FAM) was purchased from Sigma. Either 10 nM PrrF2-Cy5 or 100 nM FAM-*amiE*_6ARN_ RNA or both were incubated in ES-buffer (see above) and 25 ng tRNA in a total volume of 10 μl in the absence or presence of 120 nM Hfq, or in the absence or presence of 960 nM Crc protein. The mixtures were incubated at 37°C for 15 min to allow protein-RNA complex formation. The samples were mixed immediately before loading with 2 μl 25% glycerol or—in case no fluorescently labeled RNA was used—with 2 μl loading dye (25% glycerol, 0.2 mg/l xylencyanol and bromphenol blue), and then separated on a 4% polyacrylamide gel using Tris–borate buffer.

The radioactively or fluorescently labeled bands were visualized with a PhosphorImager (Molecular Dynamics).

## RESULTS

### Target genes of Hfq and Crc overlap

**Figure 1.**
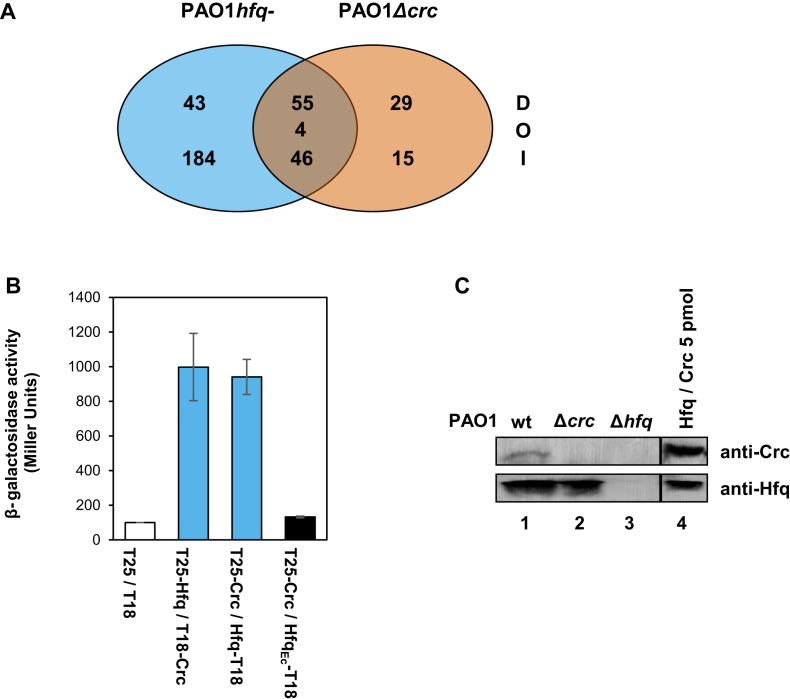
(**A**) RNA_seq_ analyses revealed an overlap between the Hfq and Crc regulon. The Venn diagram shows the number of transcripts with increased (I), decreased (D) or opposite (O) abundance in the PAO1*hfq*- and PAO1Δ*crc* mutants when compared with the PAO1 wt strain. For significance, only transcripts with a fold-change ≥5 and a multiple testing adjusted *P*-value ≤ 0.05 were considered. The corresponding transcripts with increased or decreased abundance are listed in Supplementary Table S2 (PAO1*hfq*- versus PAO1), Supplementary Table S3 (PAO1Δ*crc* versus PAO1), Supplementary Table S5 (transcripts with increased abundance affected by both, Hfq and Crc) and Supplementary Table S6 (transcripts with decreased abundance affected by both, Hfq and Crc). The transcripts showing opposite abundance (O) in the PAO1*hfq*- and PAO1Δ*crc* mutants when compared with PAO1 are listed in Supplementary Table S4. (**B**) *In vivo* association of Hfq and Crc tested with the BACTH system. N-terminal and C-terminal fusion proteins of Crc and Hfq with T18 and T25 of adenylate cyclase were constructed as described in Supplementary Text S1. The *E. coli* strain BTH101 was co-transformed with plasmids encoding the respective fusion proteins as indicated below the blue bars. Functional adenylate cyclase is only reconstituted when Crc and Hfq interact with each other, which is reflected by β-galactosidase production. White bar, background production of β-galactosidase in *E. coli* BTH101(pUT18, pKT25) harboring the parental plasmids. Black bar, co-synthesis of T25-Crc and Hfq_Ec_-T18 did not result in reconstitution of the cyclase activity. The results of three independent experiments were averaged and are shown as mean ± standard deviation. (**C**) *In vivo* co-IP of Hfq and Crc. The experiment was performed with lysates of strains PAO1 (wt) (lane 1), PAO1Δ*crc* (lane 2), and PAO1Δ*hfq* (lane 3) and anti-Hfq specific antibodies and magnetic protein G beads. The *in vivo* association of Hfq with Crc was visualized by western-blot analysis using either anti-Crc or anti-Hfq specific antibodies. The upper nitrocellulose strip was overexposed to visualize Crc. Lane 4, 5 pmol of either purified Crc or Hfq protein were loaded.

Previous studies suggested that both, Hfq and Crc, are required for tight translational repression of mRNAs, which are subjected to carbon catabolite repression (CCR) (7). Translational repression is frequently accompanied with a reduced stability and a reduced abundance of target mRNAs. Therefore, RNA_seq_ based transcriptome analyses were performed with strains PAO1, PAO1*hfq*- and PAO1Δ*crc* with the aim to reveal overlapping mRNA targets, regulation of which is governed by both, Hfq and Crc during CCR. The strains were grown to an OD_600_ of 1.5 in BSM complex medium. As succinate is the preferred carbon source of PAO1 it was included in the medium to establish CCR. The other C/N sources were added to induce transcription of the respective CCR-controlled genes (1,39). Except for glutamate, the uptake and/or utilization of the other compounds present in the BSM complex medium are known to be under CCR control (7,14,40–44). A *P*-value (adjusted for multiple testing) of 0.05 was set as a threshold for significance and only transcripts with a change in abundance (fold-change) of ±5 were considered in this study to select predominantly for transcripts that are stringently regulated during CCR. Applying these criteria 332 and 149 transcripts were found to be differentially abundant in PAO1*hfq*- and PAO1Δ*crc*, respectively, when compared with PAO1. Among these were 227 and 44 non-overlapping transcripts displaying a differential abundance in either PAO1*hfq*- or PAO1Δ*crc* when compared with PAO1 (Figure 1A; Supplementary Tables S2 and S3). In addition, four transcripts showed an opposite abundance in the PAO1*hfq*- and PAO1Δ*crc* mutants when compared with PAO1 (Figure 1A and Supplementary Table S4). Possible reasons for the seemingly independent regulation of these transcripts by either Hfq or Crc are discussed below. In addition, 105 over-lapping transcripts were found in PAO1*hfq*- and PAO1Δ*crc*, 55 and 46 of which were down-regulated and up-regulated, respectively (Figure 1A). The differential abundance of the majority of these transcripts was more pronounced in the absence of Hfq than in the absence of Crc (Supplementary Tables S5 and S6), which can be rationalized with our recent studies, wherein Hfq was shown to bind to target mRNAs and to obstruct ribosome loading, whereas Crc appeared to enhance the function of Hfq (7). When compared with PAO1, the most up-regulated transcripts in PAO1*hfq*- and PAO1Δ*crc* encode proteins involved in transport and utilization of sugars and sugar alcohols (Supplementary Table S5). Given that Hfq and Crc repress CCR-controlled genes at the translational level, this can be readily explained by translational activation of the respective genes when Hfq and Crc are absent. All transcripts with decreased abundance in PAO1*hfq*- and PAO1Δ*crc* comprise genes regulated by either of the three major quorum sensing (QS) systems LasI/R, RhlI/R and Pqs or by the quorum sensing regulator QscR (Supplementary Table S6). Given that the QS systems operate in a hierarchical manner (45) it is difficult to delineate the impact of Hfq/Crc on a particular QS system. Nevertheless, the regulatory effects of Hfq/Crc on QS are most probably indirect (46,47). Taken together, when compared with the parental PAO1 strain, the respective up-regulation of CCR-controlled genes in both the PAO1*hfq*- and the PAO1Δ*crc* strains corroborated the hypothesis that Hfq and Crc act in concert.

### *In vivo* association of Hfq and Crc

Recent *in vitro* binding studies revealed that *P. putida* Hfq, Crc and the RNA CrcZ form a complex (11). To obtain evidence for an *in vivo* interaction between Hfq and Crc, an *E. coli bac*terial *t*wo-*h*ybrid system (BACTH) was employed. In brief, we constructed various C- and N-terminal fusion proteins of PAO1 Hfq and Crc with the catalytic domains T18 and T25, respectively, of *Bordetella pertussis* adenylate cyclase. In case of an interaction between Hfq and Crc, this approach was anticipated to lead to reconstitution of functional adenylate cyclase, resulting in cAMP synthesis, which in turn is required for transcription of the *lacZ* gene. The combination of two variants of these fusion proteins, *i.e*. T25-Hfq/T18-Crc and T25-Crc/Hfq-T18, resulted in cyclase activity, indicating that Hfq and Crc interact *in vivo* (Figure 1B). All other combinations resulted in comparable low β-galactosidase activities as obtained for the control strain *E. coli* BTH101(pUT18, pKT25) (Supplementary Figure S1). Apparently, a functional interaction of the cyclase domains depended on whether the respective domains were fused to the N- or C-terminus of either Hfq or Crc. All C-terminal extensions of Crc rendered the resulting fusion proteins non-functional, indicating that C-terminal alterations in Crc might disturb the interaction with Hfq (Supplementary Figure S1). In contrast, fusions of T25 and T18 to the N- and C-terminus of Hfq, respectively, permitted an interaction with the respective N-terminal Crc fusion proteins. To ensure that endogenous *E. coli* Hfq does not interfere with the interaction of the PAO1 Hfq and Crc proteins, an *E. coli* Hfq_Ec_-T18 fusion protein was included in the assay. In contrast to T25-Crc/Hfq_Pae_-T18, co-synthesis of T25-Crc and Hfq_Ec_-T18 did not result in reconstitution of the cyclase activity, suggesting that Crc and Hfq_Ec_ do not interact in this assay (Figure 1B).

To verify that Hfq and Crc interact *in vivo* in PAO1 a co-immunoprecipitation (co-IP) experiment using anti-Hfq antibodies was performed. Anti-Hfq antibodies were added to a PAO1 lysate and then captured with magnetic protein G-beads. The beads were eluted and the proteins were resolved on SDS-polyacrylamide gels followed by detection of Hfq and Crc by western-blotting using anti-Hfq and anti-Crc antibodies, respectively. As shown in Figure 1C, lane 1, Crc was co-immunoprecipitated together with Hfq, which was not observed with lysates of the control strains PAO1Δ*crc* (Figure 1C, lane 2) and PAO1Δ*hfq* (Figure 1C, lane 3), respectively.

### The association of Hfq and Crc requires RNA bound to the distal poly(A) binding side of Hfq

**Figure 2.**
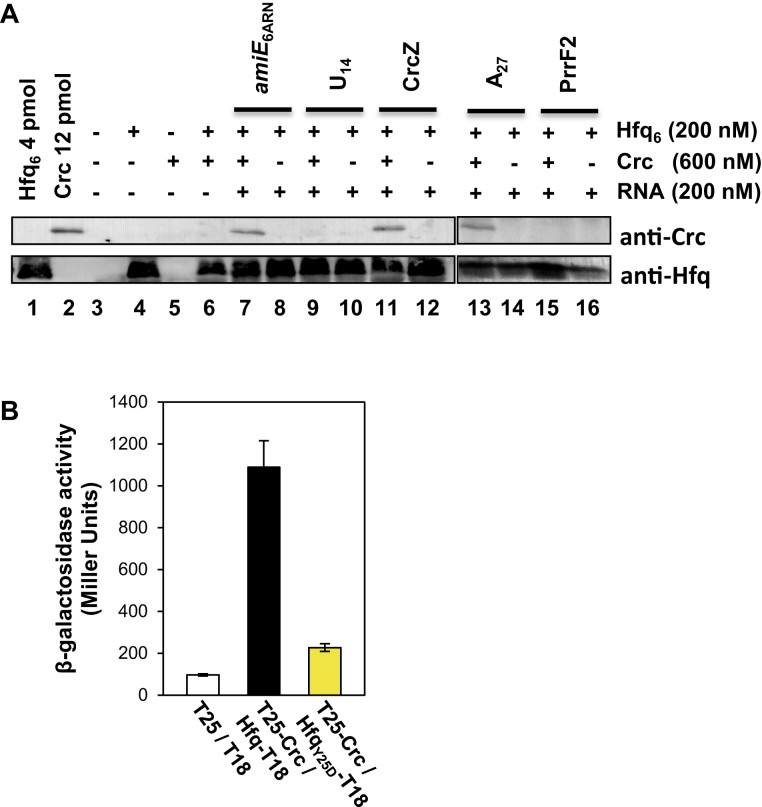
The association between Hfq and Crc requires RNA binding to the distal side of Hfq. (**A**) *In vitro* co-IP experiments were performed with purified components as indicated on top of the Figures, anti-Hfq specific antibodies and magnetic protein G beads. The *in vitro* association of Hfq with Crc was visualized by western-blot analysis using anti-Crc or anti-Hfq specific antibodies. Lanes 1 and 2, 4 pmol Hfq and 12 pmol Crc were loaded, respectively. Lanes 3–5, control experiments in the absence of both proteins (lane 3) or presence of either only Hfq (lane 4) or Crc (lane 5). Lanes 6–16, co-IP with anti-Hfq antibodies in the presence of Hfq and Crc (lanes 7, 9, 11, 13, and 15) and in the absence of Crc lanes 8, 10, 12, 14, and 16), respectively, with no RNA added (lane 6) and in the presence of *amiE*_6ARN_ (lane 7), poly-(U)_14_ (lane 9), CrcZ (lane 11), poly-(A)_27_ (lane 13) and PrrF2 sRNA (lane 15), respectively. (**B**) BACTH analysis of the Crc-Hfq_Y25D_ interaction in *E. coli* strains BTH101(pUT18, pKT25) (white bar), BTH101(pHfq-T18, pKT25-Crc) (black bar) and BTH101(pHfq_Y25D_-T18, pKT25-Crc) (yellow bar), respectively. The results of three independent experiments were averaged and are shown as mean ± standard deviation.

Crc was shown to associate with Hfq *in vitro* in the presence of CrcZ RNA (11). In contrast, no binding of Crc was observed in the presence of CrcZ with the distal side mutant Hfq_Y25D_ (11) that is defective in binding to CrcZ (7). To extend these studies, we performed *in vitro* co-IP assays with CrcZ RNA and the authentic Hfq-binding motif of the CCR-controlled *amiE* mRNA, which encodes aliphatic amidase (7). The *amiE*_6ARN_ RNA (5′-AAAAAUAACAACAAGAGG-3′) consists of six tripartite binding motifs, which can be potentially accommodated in the six distal binding pockets of Hfq (7). In addition, poly(A)_27_ RNA was used, which is likewise anticipated to bind to the distal side of Hfq (48). Moreover, poly(U)_14_ as well as PrrF2 sRNA, both of which bind to the proximal side of Hfq (14,48) were included in the assays. PAO1 Hfq and Crc were incubated in the presence of *amiE*_6ARN_ RNA, poly(U)_14_ RNA, CrcZ RNA, poly(A)_27_ or PrrF2 RNA (Figure 2A). Then, anti-Hfq antibodies were added to test whether Crc co-immunoprecipitates with Hfq using the magnetic bead technology. Without addition of RNA, Crc was not captured with Hfq (Figure 2A, lane 6). Crc associated with Hfq in the presence of either *amiE*_6ARN_ RNA (Figure 2A, lane 7), CrcZ RNA (Figure 2A, lane 11) or poly(A)_27_ RNA (Figure 2A, lane 13) but not in the presence of poly(U)_14_ RNA (Figure 2A, lane 9) or PrrF2 RNA (Figure 2A, lane 15). Taken together, these studies corroborate the hypothesis that Hfq and Crc form a complex in the presence of RNA bound to the distal side of Hfq.

To verify these *in vitro* results a Hfq_Y25D_-T18 fusion protein was included in the BACTH assay. The Y25D exchange in *P. aeruginosa* Hfq renders the protein variant defective in binding with its distal side to A-rich motifs (7). As shown in Figure 2B, the Y25D exchange in Hfq_Y25D_-T18 abolished the interaction with the T25-Crc protein. This experiment together with the *in vitro* co-IP studies supported on the one hand the idea that Hfq and Crc interact only in the presence of an RNA bound to the distal side of Hfq. Furthermore, the BACTH experiments in *E. coli* also suggested on the other hand that this interaction does not require specific RNA substrates provided that they bind to the distal side of Hfq.

**Figure 3.**
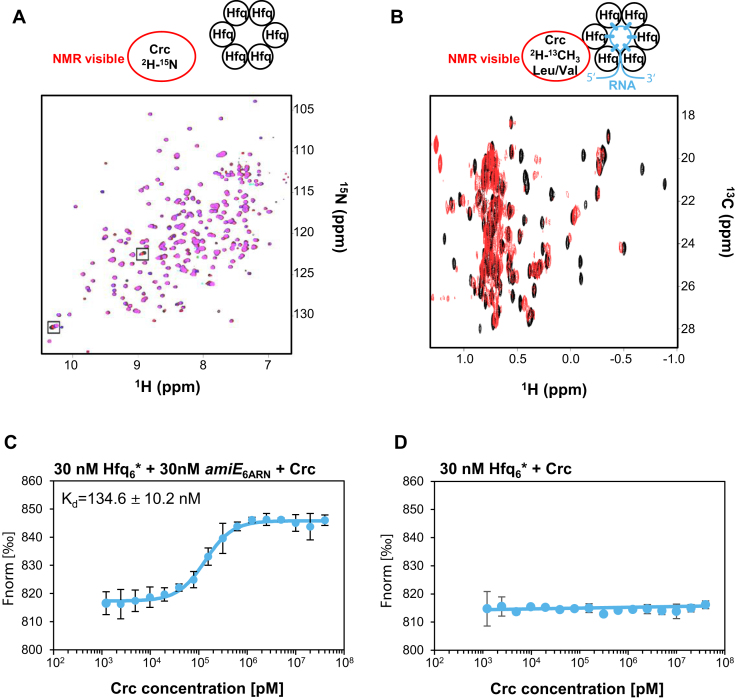
*In vitro* association between Hfq and Crc in the presence of RNA. (**A**) Overlay of the 2D ^15^N–^1^H BEST-TROSY HSQC recorded before and after addition of unlabelled Hfq_6_. The resulting spectra are colored according to the molar ratio of Crc: Hfq_6_ (black 1:0; red 1:1; blue 1:2; magenta 1:3). NMR signals that experienced chemical shift changes are boxed. (**B**) Overlay of the 2D ^13^C–^1^H HMQC spectra of ^13^C-methyl-labelled Crc recorded before (black spectra) and after addition of equimolar amounts of the unlabelled Hfq_6_/*amiE*_6ARN_ complex (red spectra). (**C**) MST analysis with 30 nM labelled Hfq_6_, 30 nM *amiE*_6ARN_ and increasing amounts of Crc. (**D**) MST analysis with 30 nM labelled Hfq_6_ and increasing amounts of Crc. Data from two independent experiments are shown as mean ± standard deviation. Thermophoresis/T-jump analysis is shown. LED power of 90% and MST power of 60% were used.

Next, solution state nuclear magnetic resonance (NMR) spectroscopy was used to analyze the interaction between Hfq and Crc in the absence and presence of RNA. As shown in Figure 3A, upon addition of increasing amounts of Hfq-hexamer (Hfq_6_) only two signals showed little chemical shift perturbations on the 2D ^1^H-^15^N HSQC spectra of ^15^N-labelled Crc. Thus, Hfq and Crc apparently hardly associate in the absence of RNA. Next, the above described *amiE*_6ARN_ RNA was incubated with six molar equivalents of unlabeled Hfq to form the Hfq-RNA assembly and used to titrate a sample of Crc with ^13^C-labeled methyl groups of leucine and valine. As shown in Figure 3B, pronounced chemical shift changes of the Crc methyl group signals were observed when both Hfq and *amiE*_6ARN_ were present and several signals experienced strong line broadening due to the high molecular weight of the formed complex. This result again indicated that Hfq and Crc associate only in the presence of RNA.

To quantitatively assess the interaction of Hfq and Crc, microscale thermophoresis (MST) was used. As shown in Figure 3C, Crc interacted with Hfq in the presence of *amiE*_6ARN_ RNA displaying a *K*_d_ of 134.6 ± 10.2 nM, whereas no detectable interaction between both proteins occurred in the absence of the RNA (Figure 3D), which concurred with the results shown in Figures 2A and 3A. As observed previously (24), Crc alone did not bind to *amiE*_6ARN_ RNA (Supplementary Figure S2), which again showed that the RNA binding protein in the Hfq/Crc/RNA complex is Hfq.

### Crc enhances the lifetime of Hfq/RNA complexes

**Figure 4.**
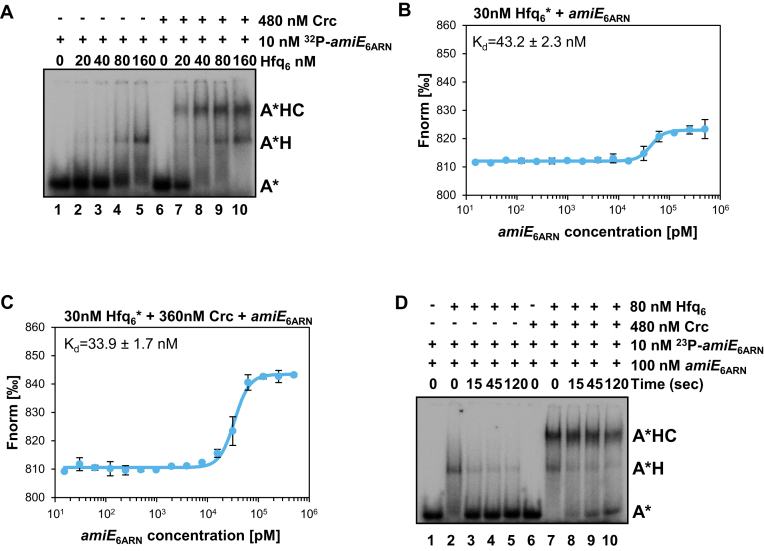
The presence of Crc stabilizes the Hfq/RNA complex. (**A**) Electrophoretic mobility shift assays (EMSA) with ^32^P-*amiE*_6ARN_ RNA and increasing amounts of Hfq_6_ in the absence and presence of Crc. Lane 1, electrophoretic mobility of ^32^P-*amiE*_6ARN_ RNA (A*) in the absence of proteins. Lanes 2–5, ^32^P-*amiE*_6ARN_ RNA/Hfq_6_ complex formation (A*H) with increasing concentrations of Hfq_6_. Lanes 6–10, ^32^P-*amiE*_6ARN_ RNA/Crc/Hfq_6_ complex formation (A*HC) with increasing concentrations of Hfq_6_. (**B** and **C**) MST analysis with 30 nM labelled Hfq_6_ and increasing concentrations of *amiE*_6ARN_ RNA in the absence (B) and presence of 360 nM Crc (C). The results represent data from two independent experiments and are shown as mean ± standard deviation. Thermophoresis/T-jump analysis is shown. LED power of 90% and MST power of 60% were used. (**D**) Hfq-RNA dissociation in the absence and presence of Crc. ^32^P-*amiE*_6ARN_ RNA was pre-incubated with Hfq (A*H complex; lanes 2–5) or with Hfq and Crc (A*HC complex; lanes 7–10). Then, unlabelled *amiE*_6ARN_ competitor RNA was added for the times given in seconds followed by electrophoresis on a native polyacrylamide gel. Lane 1, electrophoretic mobility of ^32^P-*amiE*_6ARN_ RNA (A*) in the presence of unlabelled ^32^P-*amiE*_6ARN_ RNA. Lane 6, electrophoretic mobility of ^32^P-*amiE*_6ARN_ RNA (A*) in the presence of unlabelled ^32^P-*amiE*_6ARN_ RNA and Crc. The concentrations of the ligands are given at the right.

We next studied whether the presence of Crc increases the affinity of Hfq for *amiE*_6ARN_ RNA by employing EMSA assays. The *amiE*_6ARN_ RNA was labelled at the 5′-end with [γ-^32^P]-ATP and 10 nM were incubated with increasing amounts of Hfq in the presence or absence of Crc-protein. As shown in Figure 4A, in the absence of Crc (lanes 1–5) an observable band shift was obtained when Hfq was added in 8-fold molar excess over ^32^P-*amiE*_6ARN_ RNA. In contrast, in the presence of Crc (Figure 4A, lanes 6–10) a shift of the ^32^P-*amiE*_6ARN_ RNA to the protein bound state (A*HC) occurred already when Hfq was added in 2-fold molar excess over the RNA (Figure 4A, lane 7). In addition, MST assays were performed with 30 nM labelled Hfq_6_ and increasing amounts of *amiE*_6ARN_ RNA in the absence or presence of Crc protein. The *K*_d_-value increased from 43.2 ± 2.3 nM in the absence of Crc (Figure 4B) to 33.9 ± 1.7 nM in the presence of Crc (Figure 4C). As the increase in the affinity was rather moderate this result rather argued against the idea that the presence of Crc results in a significant affinity enhancement of Hfq for the substrate.

We therefore asked whether the presence of Crc increases the stability of the Hfq/Crc/RNA complex. Pre-formed ^32^P-*amiE*_6ARN_/Hfq or ^32^P-*amiE*_6ARN_/Hfq/Crc complexes were incubated for different times with 100 nM unlabeled competitor *amiE*_6ARN_ RNA (10-fold molar excess over the labelled RNA), and then loaded after 15, 45 and 120 s on a continuously running native polyacrylamide gel. The apparent release of Hfq from the binary complex (A*H) was already observed after 15 s (Figure 4D, lane 3). In contrast, the Hfq/Crc/*amiE*_6ARN_ RNA complex was significantly more stable. Even after 120 s the majority of the labelled RNA was still present in the A*HC complex (Figure 4D, lane 10). Taken together these experiment indicated that the presence of Crc enhances the lifetime of the Hfq/RNA interaction.

### The interaction of Crc with RNA and Hfq revealed by protein-RNA and protein-protein cross-linking

**Figure 5.**
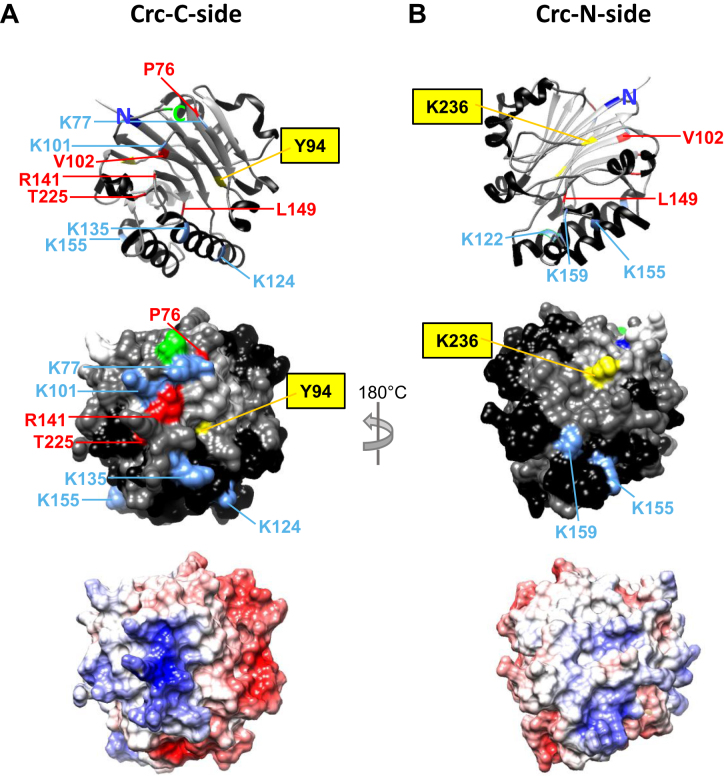
Amino acid residues in Crc implicated in Hfq and RNA interactions. The C-side and N-side of Crc are termed according to the localization of the N- and C-terminus, respectively. (A and B) Ribbon diagram (top), surface representation (middle) and electrostatic surface potential (bottom) of the Crc C-side (24) containing the basic patch (**A**) and the N-side opposed to it (**B**). The positions of the N- and C-termini are depicted in the ribbon diagrams (top) and are colored in dark blue (N-terminus) and green (C-terminus) in the surface structure (middle), respectively. K residues that were found to be cross-linked in the Hfq/Crc/*amiE*_6ARN_ complex are depicted in light blue. Residues in Crc that were found to interact with *amiE*_6ARN_ RNA are highlighted in yellow. Amino acid residues that were found to be altered in the PAO1Δ*crcZ* sup mutants are highlighted in red. α-helices are colored in black, β-strands in light grey, and coils in dark grey, respectively. Image visualization was performed with Chimera (38). The electrostatic surface potential was calculated by Coulomb's law and visualized by Chimera (38). The electrostatic potential ranges from -10 (red) to +10 (blue) kcal/(mol*e) at 298K.

A possible explanation for the RNA-induced formation of the Hfq/Crc/RNA complex was that Crc exploits multivalency as a means to stabilize Hfq/Crc/RNA complexes. In other words, even though interactions between Crc and RNA (24) and Crc and Hfq were not observed in the absence of RNA (Figures 2A and 3D), simultaneous interactions of Crc with both Hfq and RNA might considerably increase the avidity of the complex for RNA. We therefore used UV-induced cross-linking in combination with mass spectrometry to detect such interactions in the UV-cross-linked Hfq/Crc/*amiE*_6ARN_ complex. These analyses revealed that amino-acid residues Y_94_ and K_236_ in Crc interact with U/C and U bases, respectively, of the *amiE*_6ARN_ 18-mer RNA (Supplementary Figure S3A and B). Y_94_ and K_236_ are situated diametrically opposed on the surface of Crc (Figure 5A and B).

The protein-protein cross-linking experiments with bis(sulfosuccinimidyl)suberate (BS3) revealed four interactions between Crc and Hfq: Crc_K77_-Hfq_K17_, Crc_K122_-Hfq_M1_, Crc_K124_-Hfq_K3_ and Crc_K236_-Hfq_K3_, respectively (Supplementary Table S7; Figure 5A and B; Supplementary Figure S4). Hfq M_1_, K_3_ and K_17_ are situated on the proximal surface of Hfq (Supplementary Figure S4; (49)). To further validate that the Crc-Hfq interactions are only occurring in the presence of RNA, the same experiment was performed in the absence of RNA. As shown in Supplementary Table S8, no interactions between Crc and Hfq were identified under these conditions.

It was rather unexpected to detect only a few cross-links between Hfq and Crc. A most likely explanation is intrinsic to the method used. It turned out that Hfq was not well digested by trypsin and Lys-C, which probably resulted in a very limited number of Hfq-specific peptides for mass spectrometric analyses. In any case, these cross-linking experiments showed that Crc binds to RNA as well as to Hfq, indicating that the protein exploits several interactions.

In addition to the above mentioned interaction, several other BS3 induced cross-links were found originating from Crc derived peptides. The cross-links between Crc residues K_77,_ K_101_, K_124_ and K_135_ (Supplementary Table S7), which are located on the same side as the RNA binding residue Y_94_ (Figure 5A), were predominantly observed. In addition, interactions between several other Crc specific residues occurred. To distinguish between possible intra- and intermolecular interactions, the intramolecular distances between the crosslinked residues of Crc (PDP: 4JG3) were calculated with the distance calculation tool of the USCF Chimera package (Supplementary Table S7; (38)). The spacer arm length of BS3 is 11.4 Å. With the exception of the Crc_K77_-Crc_K101_ crosslink, which most likely results from the spatial proximity of both residues in Crc (24) all other cross-linked residues are further apart (Supplementary Table S7), which favors the idea that the observed Crc-Crc interactions are intermolecular. In addition, a number of apparent intermolecular Crc-Crc cross-links were also noticeable when BS3 cross-linking was performed with Hfq and Crc in the absence of RNA (Supplementary Table S8). However, they occurred to a lower extend when compared with those observed in the Hfq/Crc/*amiE*_6ARN_ complex. There is so far no evidence for oligomerization of Crc monomers from structural (24) and biochemical studies (Supplementary Figure S5C). Thus, we can only speculate that weak interactions occurred under these experimental conditions, which are not revealed by other means.

These studies raised the question whether the size of the Hfq/Crc/RNA complex is larger than expected form a 1:1:1 stoichiometry. The size of the complex was assessed by SEC-MALS (size exclusion chromatography combined with multi-angle laser light scattering; Supplementary Text S1). In solution, the measured molecular mass of the Hfq/Crc/*amiE*_6ARN_ complex was determined with 219 800 g mol^−1^ (Supplementary Figure S5A). Given that the Hfq hexamer and the Crc monomer are 53.7 kDa (Supplementary Figure S5B) and 29.6 kDa (Supplementary Figure S5C) proteins in solution, respectively, and that the *amiE*_6ARN_ RNA accounts for ∼ 6100 g mol^−1^, the size of the complex indicates that several Hfq and/or Crc molecules are present. The presence of at least two Crc monomers in the Hfq/Crc/*amiE*_6ARN_ complex is indicative from some cross-links obtained with BS3 as the Crc aa residues K_77_, K_135_, K_155_, and K_236_ were found to be auto-linked (Supplementary Table S7).

### Genetic dissection of the Hfq-Crc interaction

To gain further insights into the Hfq-Crc interactions, we made use of the observation that a PAO1Δ*crcZ* strain is defective in utilization of a number of carbon sources (Supplementary Table S9). This can be explained by the absence of the CrcZ preventing relief of Hfq/Crc-mediated repression of gene functions required for metabolisation of these carbon sources (7). PAO1Δ*crcZ* revertants that regained the ability to grow on either histidine, alanine, acetamide or mannitol were screened for mutations in the *hfq* and *crc* genes with the rationale to isolate variants that lost the ability to interact with either Hfq or Crc. The PAO1Δ*crcZ* revertants were analyzed by means of colony PCR followed by DNA sequencing of the *hfq* and *crc* genes (Supplementary Text S1). This analysis revealed 25 intragenic mutations in the *crc* gene, one in the *hfq* gene and two extragenic suppressor mutations, the latter of which were not further characterized (Supplementary Table S10).

The mutation in the *hfq* gene resulted in an exchange of P_64_ to S (Supplementary Table S10). As shown in Supplementary Figure S4A and B, P_64_ is located proximal to the flexible C-terminus and could affect its lateral orientation (49–51).

Among the twenty-five mutations detected in the *crc* coding region were such that led to the generation of premature stop codons, deletions, frame-shifts or to an extension of the reading frame (Supplementary Table S10). These were not further studied. Six missense mutations resulting from single nucleotide changes in *crc* were identified, which resulted in amino acid substitutions at five different positions in Crc (Supplementary Table S10). Three (P_76_, R_141_, T_225_) of the five altered amino acid residues are located on the C-side of Crc (Figure 5A), and are fully conserved in the Crc proteins of the sequenced *Pseudomonadaceae* (not shown). V_102_ is close to the surface and in the same region as the other surface exposed residues, whereas L_149_ is buried inside the globular structure of Crc (Figure 5A and B). V_102_ and L_149_ are not fully conserved in the Crc proteins of the *Pseudomonadaceae* but the variations comprise amino-acid residues with strongly similar properties (not shown). It is also worth noting that K_101_ and K_77_, which were frequently found to cross-link with other Crc residues (Supplementary Table S7), as well as Y_94_, which was cross-linked to RNA, are as well located in the same region on the Crc surface (Figure 5A and B).

For further analysis we focused on the following revertants: PAO1Δ*crcZ*_sup34_ (Hfq_P64S_), PAO1Δ*crcZ*_sup2b7_ (Crc_V102E_), PAO1Δ*crcZ*_sup29_ (Crc_L149R_), PAO1Δ*crcZ*_supA_ (Crc_P76L_), PAO1Δ*crcZ*_supE_ (Crc_T225I_) and PAO1Δ*crcZ*_supG_ (Crc_R141L_). Since the PAO1Δ*crcZ* revertants were isolated after growth on different carbon-sources, we first tested whether their phenotype was independent on the respective carbon source used for their selection. All mutants were able to grow on histidine, alanine, acetamide and mannitol (Supplementary Figure S6). We therefore hypothesized that the respective Hfq and Crc variants lead to a general alleviation of CCR.

To corroborate this, we tested the proficiency of the Hfq and the Crc mutant proteins to repress translation of an *amiE::lacZ* reporter gene during CCR (7). The strains PAO1, PAO1Δ*crcZ*, PAO1Δ*hfq*, PAO1Δ*crc* and the six PAO1Δ*crcZ* mutant strains were transformed with plasmid pME9655, encoding a translational *amiE::lacZ* fusion gene, and with the empty vector pME4510 (Supplementary Table S11). The strains were subjected to CCR by cultivation in BSM medium supplemented with 40 mM succinate and 40 mM acetamide, the latter of which was added to induce transcription of the *amiE::lacZ* gene. When compared with the PAO1Δ*crcZ* (pME9655, pME4510) strain, the translation of the *amiE::lacZ* reporter gene was increased in all PAO1Δ*crcZ* revertants, albeit to a lower level when compared with the PAO1Δ*crc*(pME9655, pME4510) and PAO1Δ*hfq*(pME9655, pME4510) strains (Supplementary Table S11). This indicated a partial loss of function of the Crc variants and of the Hfq_P64S_ mutant protein with regard to Hfq/Crc/RNA complex formation. The complementation of the *crc* and *hfq* alleles of the PAO1Δ*crcZ* revertants with plasmid encoded wild-type copies of *crc* (pME4510*crc*_Flag_) and *hfq* (pME4510*hfq*_Flag_) (Supplementary Table S11), respectively, resulted in full repression of *amiE::lacZ* translation, which clearly attributed the PAO1Δ*crcZ* suppressor phenotype to the *crc* and *hfq* missense alleles. On the other hand, it suggested that the relief of Hfq/Crc-mediated repression observed with the revertants (Supplementary Table S11) is attributable to an impairment in the Hfq/Crc/RNA interaction.

Next, the BACTH assay was employed to further test whether the mutant proteins encoded by the different *crc* and *hfq* missense alleles are impaired in Hfq/Crc interactions. As shown in Supplementary Figure S7, none of the T25-Crc variant proteins interacted with Hfq-T18. In addition, the interaction of Hfq_P64S_-T18 with T25-Crc was apparently impaired. Taken together, these experiments indicated that the respective Hfq and Crc variants are defective in Hfq/Crc complex formation, which can readily explain the observed alleviation of CCR in the PAO1Δ*crcZ* revertants.

As discussed below, although we cannot distinguish whether these single amino acid changes affect binding of Crc to Hfq or RNA, it seems worth noting that they are located in close proximity to Y_94_, which cross-linked with RNA (Figure 5A). The Hfq_P64S_ mutant protein was apparently impaired in Hfq/Crc/RNA complex formation (Supplementary Table S11) and Crc cross-linked to Hfq K_3_ and K_17_. The latter residues are part of the N-terminal α-helix situated on the proximal side of Hfq (Supplementary Figure S5A), and, as mentioned above, Hfq_P64_ is located proximal to the flexible C-terminus and most likely affects its lateral orientation on the proximal side. Therefore, we next asked whether Crc might interfere with binding of a sRNA to the proximal side of Hfq.

### Crc interferes with binding of a sRNA to the proximal side of Hfq

**Figure 6.**
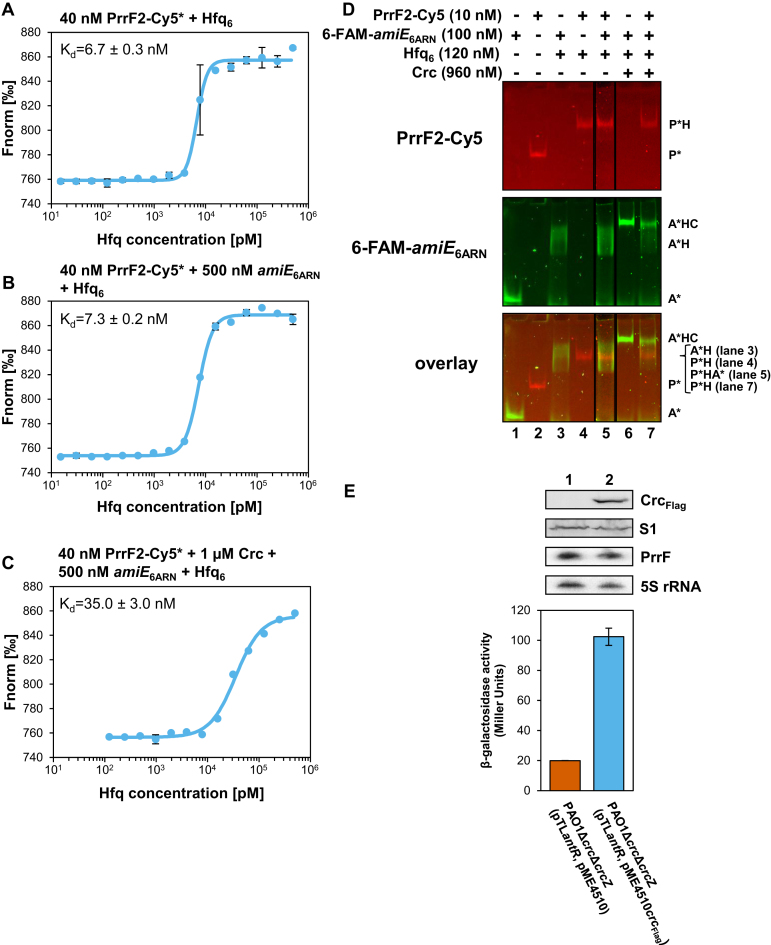
Crc affects binding of the sRNA PrrF2 to Hfq. MST analyses with 40 nM PrrF2-Cy5 RNA and (**A**) increasing concentrations of Hfq, (**B**), increasing concentrations of Hfq in the presence of 500 nM *amiE*_6ARN,_ and (**C**) increasing concentrations of Hfq in the presence of 500 nM *amiE*_6ARN_ and 1 μM Crc. The results represent data from two independent experiments and are shown as mean ± standard deviation. Thermophoresis/T-jump analysis is shown. LED power of 90% and MST power of 60% were used. (**D**) PrrF2 sRNA does not bind to the Hfq/Crc/*amiE*_6ARN_ RNA complex. EMSA with 10 nM Cy5-labelled PrrF2 RNA (red bands) and 100 nM 6-FAM-labelled *amiE*_6ARN_ RNA (green bands) in the absence or presence of Hfq_6_ and Crc. Lane 1, electrophoretic mobility of 6-FAM-*amiE*_6ARN_ RNA (A*). Lane 2, electrophoretic mobility of PrrF2-Cy5 RNA (P*). Lanes 3 and 4, electrophoretic mobility of 6-FAM-*amiE*_6ARN_ RNA (A*H; middle panel) and PrrF2-Cy5 RNA (P*H; upper panel), respectively, in the presence of 120 nM Hfq. Lane 5, electrophoretic mobility of PrrF2-Cy5 RNA and 6-FAM-*amiE*_6ARN_ RNA in the presence of 120 nM Hfq. As shown in the superimposition (bottom panel) both RNAs are in complex with Hfq (P*HA*). Lane 6, electrophoretic mobility of 6-FAM-*amiE*_6ARN_ RNA in the presence of 120 nM Hfq and 960 nM of Crc (A*HC). Lane 7, electrophoretic mobility of PrrF2-Cy5 RNA and 6-FAM-*amiE*_6ARN_ RNA in the presence of both, 120 nM Hfq and 960 nM Crc. As shown in the superimposition PrrF2-Cy5 RNA is not part of the Hfq/Crc/6-FAM-*amiE*_6ARN_ (A*HC) RNA complex. Only the Hfq bound state (P*H) is observed. (**E**) The strains PAO1Δ*crc*Δ*crcZ*(pTL*antR*, pME4510) (orange bar) and PAO1Δ*crcΔcrcZ*(pTL*antR*, pME4510*crc*_Flag_) (blue bar) were grown in BSM-succinate medium. Samples were withdrawn at an OD_600_ of 2.0. The bars represent the β-galactosidase values conferred by the plasmid pTLantR encoded translational *antR::lacZ* fusion in the presence or absence of ectopic *crc*_Flag_ expression, respectively. The error bars represent standard deviations from three independent experiments. Top panel, Crc_Flag_, S1, PrrF2 and 5S rRNA levels in strains PAO1Δ*crc*Δ*crcZ*(pTL*antR*, pME4510) (lane 1) and PAO1Δ*crcΔcrcZ*(pTL*antR*, pME4510*crc*_Flag_) (lane 2). The Crc_Flag_ levels were determined by western-blot analysis using anti-Crc antibodies. Immuno-detection of ribosomal protein S1 served as a loading control. The PrrF2 and 5S rRNA (control) levels were determined by Northern blotting.

To address this, we made use of our recent observation that the PAO1 sRNA PrrF1–2 binds to the proximal side of Hfq (14). First, MST was used to test whether the presence of Crc interferes with PrrF2 binding. As shown in Figure 6A, the K_d_ of Hfq for Cy5 labelled PrrF2 was determined with ∼ 6.7 ± 0.3 nM. The K_d_ of Hfq for PrrF2-Cy5 did not significantly change in the presence of a 12-fold molar excess of *amiE*_6ARN_ RNA (Figure 6B), the latter of which was shown to bind to the distal side of Hfq (7). However, when Crc was additionally included in the assay, the *K*_d_ of Hfq for PrrF2-Cy5 declined to 35.0 ± 3.0 nM (Figure 6C), indicating that the formation of the Hfq/Crc/*amiE*_6ARN_ complex interferes with binding of the sRNA to Hfq. Next, EMSA assays were performed with PrrF2-Cy5 and *amiE*_6ARN_FAM in the presence of Hfq as well as in the presence of both, Hfq and Crc. As shown in Figure 6D, lane 5, in the presence of Hfq alone, both RNAs were bound to Hfq. When Crc was additionally included in the assay, a supershifted species of Hfq/Crc/6-FAM-*amiE*_6ARN_ was observed, but a quaternary complex composed of Hfq/Crc/6-FAM-*amiE*_6ARN_/PrrF2-Cy5 was not observed (Figure 6D, lane 7). Although the latter experiment might be interpreted as showing that Crc interferes with PrrF2 binding to the Hfq/Crc/6-FAM-*amiE*_6ARN_ complex, the experimental set up does not exclude the possibility that Hfq binds to either *amiE*_6ARN_ or PrrF2.

Therefore, we sought to verify this observation *in vivo* by asking whether Crc can interfere with Hfq-mediated and PrrF1–2 dependent translational repression of *antR* mRNA (14). The β-galactosidase activity conferred by a translational *antR::lacZ* reporter gene expressed from plasmid pTL*antR* was monitored in strain PAO1Δ*crc*Δ*crcZ*. As both Hfq and Crc were shown to impact on the level of CrcZ RNA (52), the double mutant was chosen to exclude the possibility that CrcZ interferes with Hfq-mediated and PrrF1–2 dependent riboregulation of *antR* mRNA (14). In addition to plasmid pTL*antR*, the strain harbored either the parental vector pME4510 or plasmid pME4510*crc*_Flag_, which permitted over-production of a Crc_Flag_ variant (Figure 6E top panel, lane 2). When compared with strain PAO1Δ*crc*Δ*crcZ*(pTL*antR*, pME4510), over-expression of *crc*_Flag_ in strain PAO1Δ*crc*Δ*crcZ*(pTL*antR*, pME4510*crc*_Flag_) resulted in de-repression of *antR::lacZ* translation (Figure 6E). As the *antR* promoter activity was unaffected by Crc under these conditions (Supplementary Figure S8), this observation agreed with the notion that Crc can interfere with binding of PrrF2 to the proximal side of Hfq. The RNA_seq_ analyses lend support to these observation. They revealed four transcripts that showed an opposite abundance in PAO1*hfq*- and PAO1Δ*crc* when compared with PAO1. Among them are the *antABC* transcripts, (Supplementary Table S4), which are positively regulated by the transcription factor AntR. These genes were up-regulated in PAO1*hfq*-, consistent with the finding that negative translational regulation of the *antR* mRNA by the sRNA PrrF1–2 is abrogated in the absence of Hfq (14). In opposite, the *antABC* transcripts showed a decreased abundance in PAO1Δ*crc*. This in turn can be reconciled with the experiment shown in Figure 6E, sindicating that translational repression of *antR* by Hfq and PrrF1–2 is more efficient in the absence of Crc.

## DISCUSSION

### The Hfq/Crc/RNA complex, a multipart ensemble

Genetic, biochemical and biophysical studies showed that efficient Hfq/Crc/RNA complex formation requires binding of an RNA molecule to the distal poly(A) binding side of Hfq (Figures 2 and 3). We infer from the BACTH assays carried out in *E. coli* (Figure 1B), that the nature of the distal bound RNA does not matter provided that it contains an A-rich recognition motif for Hfq. The bound RNA could bring about the Hfq/Crc interaction by inducing conformational changes that permit the interaction with Crc. Although there is some evidence from NMR studies for a cross-talk between the distal and proximal sides of Hfq upon poly(A) binding (53), other biophysical studies suggested that the core region of *E. coli* Hfq is rather rigid (48,51). Given that Crc was shown to cross-link with both, Hfq and RNA, we rather favor the idea that Crc exploits several interactions as a means to assemble into Hfq/Crc/RNA complexes. As Crc does not detectably interact with Hfq alone (Figure 3D), and the affinity of Hfq is comparable to that of the Crc/Hfq/RNA complex for the distal bound RNA (Figure 4B and C), we hypothesize that the RNA in the Hfq-RNA complex serves as a toehold for Crc assembly. Although the presence of Crc did not significantly enhance the affinity of Hfq for the RNA (Figure 4C), the simultaneous interactions of Crc with both binding partners result in an Hfq/Crc/RNA assembly with increased lifetime when compared with the Hfq/RNA complex alone (Figure 4D). This in turn can explain the function of Crc in Hfq-mediated translational repression of target mRNAs during CCR (7,11). Hence, Crc is the first proteinaceous factor shown to modulate Hfq-mediated RNA binding.

The intermolecular distances between the cross-linked and surface exposed K residues in Crc as well as between the auto-linked K residues 77, 135, 155 and 236 (Supplementary Table S7) are indicative for intermolecular interactions between Crc entities, which is in agreement with the observation that Hfq, Crc and RNA do apparently not assemble with a 1:1:1 stoichiometry (Supplementary Figure S5). It is also worth noting that the *crc* suppressor mutations affecting amino acid residues 76 (P_76_L) and 102 (V_102_E) are in juxtaposition to K_77_ and K_101_ that were found to cross-link frequently with Crc residues 135/155 and 135, respectively (Supplementary Table S7). In addition, Crc_K77_ cross-linked with Hfq_K17_. Thus, the loss of function of these mutant proteins with regard to the Hfq/Crc mediated repression of *amiE::lacZ* translation (Supplementary Table S11) and Hfq/Crc interaction (Supplementary Figure S7) could result from their negative effect on both Hfq/Crc and Crc/Crc interactions.

As we have identified only two cross-linked amino-acids on the opposite sides of Crc that interacted with RNA (Figure 5A and B), it is not possible to delineate a RNA binding surface. In addition, as *amiE*_6ARN_ RNA contains only one U-nucleotide, we cannot distinguish whether two RNA molecules are bound on either side of Crc or whether they are bound to two Crc proteins. The electrostatic surface potential of Crc revealed a basic patch on the C-side of the protein (Figure 5A, bottom). Crc Y_94_ that cross-linked with *amiE*_6ARN_ RNA as well as the amino acid exchanges T_225_I R_141_C/S identified in the genetic screen (Supplementary Table S10) are in close proximity or within this basic patch. The C-terminus of Crc is situated on top of the basic patch. The *crc* sup2a4 mutation altered the C-terminus in that it resulted in a 21 amino acid extension (Supplementary Table S10). Similarly, C-terminal extensions with either the T18 or the T25 domain of the adenylate cyclase rendered Crc inactive in interacting with Hfq in the BACTH system (Supplementary Figure S1). On the other hand, one of the most prominent Crc–Crc intermolecular cross-links (K_101_/K_135_) is found in this region. Hence, it seems worthwhile to further address the question whether the C-side is involved in RNA binding as well as in Crc–Crc interactions.

Only four intermolecular cross-links, Hfq_M1_-Crc_K122_, Hfq_K3_-Crc_K124,_ Hfq_K3_-Crc_K236,_ and Hfq_K17_-Crc_K77_ were obtained between Hfq and Crc. (Figure 5; Supplementary Figure S4; Supplementary Table S7). As mentioned above, this can most likely be attributed to the stability of Hfq in the presence of the proteases trypsin/Lys-C. Hfq also displayed complete resistance to the proteases pepsin and chymotrypsin. Thus, there may be more interactions between both proteins that escaped our analyses. M_1_, K_3_ and K_17_ are located on the proximal site of Hfq (Supplementary Figure S4). The *hfq* sup34 mutation resulted in an exchange of P_64_ to S. Hfq_P64_ represents the last amino acid of the conserved core of Hfq and precedes the C-terminus, which is most likely intrinsically unstructured (51). The C-terminus of Hfq seems to extend laterally away from the proximal side of Hfq (51,54,55). Thus, the Hfq_P64_ exchange might impact on the spatial orientation of the C-terminus and thus affect Crc binding. In any case, (i) RNAs binding to the proximal side did not result in Hfq/Crc/RNA complex formation (Figure 2A), (ii) a quaternary Hfq/Crc/6-FAM-*amiE*_6ARN_/PrrF2-Cy5 complex was not observed (Figure 6D), and (iii) Crc apparently interfered with Hfq-mediated and PrrF1–2-dependent riboregulation of *antR* mRNA. We interpret these results as showing that PrrF1–2 and Crc binding to Hfq is exclusive. Whether this also holds for other sRNAs remains to be studied. Currently efforts are underway to elucidate the composition and architecture of the Hfq/Crc/RNA ensemble by means of structural biology.

### Physiological implications for modulation of Hfq function by Crc

The observed differences in the transcriptomes between wild-type strains and the isogenic *hfq* deletion mutants grown in different media ((46,57); PRJEB22802) established Hfq as a pleiotropic regulator in *Pseudomonas* impacting on metabolism (7), establishment of virulence traits (10,56,57) including quorum sensing (46,47) as well as on certain stress responses (58). As anticipated from a concerted action of Hfq and Crc, the transcriptome analyses revealed an overlap between the Hfq and the Crc regulon (Supplementary Tables S5 and S6). However, given the criteria used for the RNA_seq_ analysis a number of non-overlapping transcripts displayed a differential abundance in either PAO1*hfq*- or PAO1Δ*crc* when compared with PAO1 (Figure 1A; Supplementary Tables S2 and S3). Here, the Hfq regulon is larger than the Crc regulon (Figure 1A). This might be explained in light of the multiple tasks of Hfq, which also involves canonical riboregulation with sRNAs (14,15). Therefore, it may not be surprising that the genetic screen did not reveal several *hfq* suppressor mutants. On the hand, the opposite abundance of transcripts in PAO1*hfq*- and PAO1Δ*crc*, as exemplified by the *antABC* transcripts (Supplementary Table S4), most likely results from the interference of Crc with Hfq and sRNA-mediated riboregulation. Moreover, Crc is apparently involved in regulating a sub-set of Hfq regulated genes (Figure 1A), which might explain the higher number of suppressor mutations found in the *crc* gene. However, it should also be noted that for significance only transcripts with a fold-change of ± 5 were considered. By lowering this threshold the number of overlapping genes was obviously increasing (not shown). Nevertheless, further efforts are necessary to understand how Crc impacts on transcripts that are not concurrently subject to regulation by Hfq.

A puzzling aspect of the study is that the affinity of Hfq for the sRNA PrrF2 is higher (Figure 6A) than the affinity of Crc for the Hfq/*amiE*_6ARN_ complex. (Figure 3C). We hypothesize that this ensures that sRNA-mediated regulation of stress responses is put into effect when required, while the increased stability of the Hfq/Crc/RNA complexes (Figure 4D) permits maintenance of CCR, *i.e*. it would safeguard that Crc prioritizes the function of Hfq toward optimal carbon utilization. This working model would require more free Hfq than Crc in the cell. We have argued that in the presence of a preferred carbon source only a few other catabolites may induce concomitant transcription of the corresponding catabolic genes (7). In addition, translational repression during CCR of catabolic genes other than those required for the breakdown of the preferred carbon source appears to lead to degradation of the corresponding mRNAs (7), and thus most likely to recycling of Hfq. Therefore, CCR control may not require vast amounts of Hfq. The intracellular concentration of Hfq has been calculated with ∼2160 ± 56 Hfq_6_ during growth in BSM-succinate medium at an OD_600_ of 2.0 (7). This is comparable with ∼2350 ± 481 Crc monomers per cell determined under the same conditions (Supplementary Figure S9). The Crc levels appear not to vary with growth phase or with the carbon source (8), which indicates that Hfq_6_ and Crc-monomers are present in ∼ equimolar quantities in the cell. However, given the size of the Hfq/Crc/RNA complex (Supplementary Figure S5A) and the number of intermolecular cross-links observed for Crc (Supplementary Table S7) one might speculate that the Hfq/Crc/RNA complex involves more Crc-monomers than Hfq_6_. In such a scenario enough Hfq_6_ might be free for stress-induced riboregulation with sRNAs during CCR.

## Supplementary Material

Supplementary Data
