# Supplementary material for: Interplay between the catabolite repression control protein Crc, Hfq and RNA in Hfq-dependent translational regulation in Pseudomonas aeruginosa
Source: Nucleic Acids Res. 2017 Dec 13;46(3):1470–85. doi: 10.1093/nar/gkx1245 (PMC5815094; doi:10.1093/nar/gkx1245)
Supplement: Supplementary Data [file gkx1245_supp.pdf]

TEXT S1 SUPPLEMENTARY INFORMATION

Supplementary Materials and Methods

Construction of plasmids. Plasmid pME4510crc$_{\mathbf{Flag}}$. To construct a *crc*$_{\text{Flag}}$ fusion gene under control of its authentic promoter, a 906-base pair (bp) fragment of *crc* (nt -129 to nt +777 with regard to the A (+1) of the start codon) was amplified by PCR using oligonucleotides W74 (5´-TTT TTT GAA TTC CCC CGC GCT CGA TGG C-3´) and Z74 (5´-TTT TCT GCA Gtc act tgt cgt cat cgt ctt tgt agt cGA TGC TCA ACT GCC AGT C-3´) and chromosomal DNA as template. Primer Z74 contains a Flag-tag sequence (lower case letters); restriction sites are marked in bold. The PCR fragment was cleaved with EcoRI and PstI and ligated into the corresponding sites of plasmid pME4510.

Plasmids pKT25hfq, pKT25crc, pKT25crc$_{\mathbf{L149R}}$, pKT25crc$_{\mathbf{V102E}}$, pKT25crc$_{\mathbf{R141C}}$, pKT25crc$_{\mathbf{T225I}}$ and pKT25crc$_{\mathbf{P76L}}$. For construction of the N-terminal fusions proteins between the T25 domain of CyaA (corresponds to *cyaA* nt +1 to nt +672 with regard to the A (+1) of the start codon) and Hfq, Crc or the respective mutant proteins, a 300-bp fragment of *hfq* (nt -2 to nt +298 with regard to the A (+1) of the start codon) and a 848-bp fragment of *crc* (nt -2 to nt +846 with regard to the A (+1) of the start codon) was amplified by PCR using oligonucleotides E120 (5´-TTT TTT TCT GCA GAC ATG TCA AAA GGG CAT TCG CTA C-3´) and F120 (5´-TTT TTT TGA ATT CCA GCC TGT TCC CCA CCA CC-3´) for *hfq* and T117 (5´-TTT TTT TCT GCA GTT ATG CGG ATC ATC AGT GTG AAC-3´) and U117 (5´-TTT TTT TGA ATT CTC AAG TAG GCC GCA CGG GAA-3´) for *crc* and chromosomal DNA of PAO1, PAO1Δ*crcZ*$_{\text{sup29}}$, PAO1Δ*crcZ*$_{\text{sup2b7}}$, PAO1Δ*crcZ*$_{\text{supE}}$, PAO1Δ*crcZ*$_{\text{supG}}$ and PAO1Δ*crcZ*$_{\text{supA}}$, respectively. The respective PCR fragments were cleaved with EcoRI and PstI and ligated into the corresponding sites of plasmid pKT25.

Plasmids p25-N*hfq*, p25-N*crc*. For construction of the C-terminal fusions proteins between the T25 domain of CyaA (corresponds to *cyaA* nt +1 to nt +672 with regard to the A

(+1) of the start codon) and Hfq or Crc, a 246-bp fragment of *hfq* (nt -1 to nt +245 with regard to the A (+1) of the start codon) and a 777-bp fragment of *crc* (nt -1 to nt +776 with regard to the A (+1) of the start codon) was amplified by PCR using oligonucleotides V117 (5´-TTT TTT TCT GCA GCA TGT CAA AAG GGC ATT CGC TAC-3´) and W117 (5´-TTT TTT TGA ATT **C**GC GTT GCC CGG CTC GGC C-3´) for *hfq* and C120 (5´-TTT TTT TCT GCA GTA TGC GGA TCA TCA GTG TGA AC-3´) and D120 (5´-TTT TTT TGA ATT CAT GCT CAA CTG CCA GTC GTA G-3´) for *crc* and chromosomal DNA of PAO1. The respective PCR fragments were cleaved with EcoRI and PstI and ligated into the corresponding sites of plasmid p25-N.

Plasmids pUT18*hfq*, pUT18*hfq*$_{\mathbf{Ec}}$, pUT18*crc*, pUT18*hfq*$_{\mathbf{P64S}}$ and pUT18*hfq*$_{\mathbf{Y25D}}$. For construction of the C-terminal fusions between the T18 domain of CyaA (corresponds to cyaA nt +673 to nt +1197 with regard to the A (+1) of the start codon) with PAO1 Hfq, *E. coli* Hfq and Crc, a 246-bp fragment of PAO1 *hfq* (nt -1 to nt +245 with regard to the A (+1) of the start codon), respectively, a 310-bp fragment of *hfq*$_{Ec}$ (nt -1 to nt +309 with regard to the A (+1) of the start codon) as well as a 777-bp fragment of *crc* (nt -1 to nt +776 with regard to the A (+1) of the start codon) were amplified by PCR using oligonucleotides V117/W117 (see above) for *hfq* and Y122 (5´-TTT TTT TCT GCA GAA TGG CTA AGG GGC AAT CTT TAC-3´) and Z122 (5´-TTT TTT TGA ATT CTC GGT TTC TTC GCT GTC CTG TTG-3´) for *hfq*$_{Ec}$ and V117 (see above) and C120/D120 (see above) for *crc* and either chromosomal DNA of PAO1 or *E. coli* MC4100F'. For the T18 fusions with the PAO1 Hfq$_{P64S}$ and PAO1 Hfq$_{Y25D}$ variants, the PCR fragments were obtained with oligonucleotides V117/W117 (see above) and chromosomal DNA of PAO1Δ*crcZ*$_{supp34}$ and DNA of plasmid pME4510*hfq*$_{Y25D}$, respectively. The respective PCR fragments were cleaved with EcoRI and PstI and ligated into the corresponding sites of plasmid pUT18.

Plasmids pUT18C*hfq* and pUT18C*crc*. For construction of N-terminal fusions between the T18 domain of CyaA (corresponds to *cyaA* nt +673 to nt +1197 with regard to the A (+1) of the start codon) with PAO1 Hfq and Crc, a 299-bp fragment of *hfq* (nt -1 to nt +298 with regard to the A (+1) of the start codon) and a 847-bp fragment of *crc* (nt -1 to nt +846 with regard to the A (+1) of the start codon) were amplified by PCR using oligonucleotides X120 (5´-TTT TTT TCT GCA GCA TGT CAA AAG GGC ATT CGC TAC-3´) and F120 (see above) for *hfq* and W120 (5´-TTT TTT TCT GCA GTA TGC GGA TCA TCA GTG TGA AC-3´) and U117 (see above) for *crc* and chromosomal DNA of PAO1. The respective PCR fragment were cleaved with EcoRI and PstI and ligated into the corresponding sites of plasmid pUT18C.

Plasmid pET26bII-Crc. A 777 bp fragment of *crc* (nt +1 to nt +777 with regard to the A (+1) of the start codon) was amplified by PCR using oligonucleotides 5´-ATA TAC ATA TGC GGA TCA TCA GTG TGA ACG-3´ and 5´- TAT ATC TCG AGG ATG CTC AAC TGC CAG TCG-3´ and chromosomal DNA of PAO1. The PCR fragment was then cleaved with NdeI and XhoI and ligated into the corresponding sites of plasmid pET26bII.

Plasmid pME9679. To construct a PAO1 in frame chromosomal deletion mutant in the *crcZ* gene the following procedure was used. First, two PCR products were obtained using primer pairs U1 (5´-ACG T**GG ATC C**AC CGC GAC CTG AAA ACC C-3´)/V1 (5´-CGG TGG GTC GGC GGA GGG CAC-3´) and W1 (5´-GTG CCC TCC GCC GAC CCA CCG ACT TGG GGG GGA GCT TCG G-3´)/X1 (5´-ACG T**GA ATT C**GG CGC GGA CCT GC-3´) and chromosomal DNA of PAO1, respectively. The annealed 666-bp upstream and 659-bp downstream fragments were used as a template for a second overlapping PCR with primers U1 and X1 (V1 and W1 contain a complementary sequences). The resulting fragment - with a 408-bp deletion in *crcZ*, which spans the promoter region of *crcZ* and the majority of the *crcZ*

gene (nucleotides 5308523- 5308930 (59)) - was cleaved with BamHI and EcoRI and ligated into the corresponding sites of the suicide vector pME3087.

Construction of strains PAO1Δ*crcZ*$_{\mathbf{ab}}$ (PAO6713) and PAOΔ*crc*Δ*crcZ*. The strain PAO6713 and the PAOΔ*crc*Δ*crcZ* double mutant were constructed by homologous recombination. Briefly, plasmids pME9679 (for PAO6713) and plasmid pME9673 (for PAOΔ*crc*Δ*crcZ*) were mobilized into strain PAO1 and PAO6673, respectively, with the aid of *E. coli* strain HB101(pRK2013), and then chromosomally integrated through selection for tetracycline resistance. Excision of the vector by a second crossover event was achieved by enrichment for tetracycline-sensitive cells (60).

Protein purification. Hfq protein was produced in the *hfq* deficient *E. coli* strain AM111F′ harbouring plasmid pHfq$_{\mathrm{Pae}}$. The protein was purified as described in Beich-Frandsen *et al.* (51).

The Crc protein was purified from *E. coli* strain BL21(DE3)(pETM14lic-His$_6$Crc) by Ni-affinity chromatography, followed by removal of the His$_6$-tag with GST-HRV14-3C ‘‘PreScission’’ protease as described by Milojevic *et al.* (24).

SEC-MALS. Hfq protein with 0.98 absorbance at 280 nm was applied to SEC-MAL superdex 10/300 GL column, equilibrated with a buffer containing 50 mM Tris-HCl pH 7.2, 200 mM NaCl, 2 mM TCEP. Crc protein with 0.37 absorbance at 280 nm was applied to superdex 10/300 GL column, equilibrated with a buffer containing 50 mM HEPES, 150 mM NaCl. The complex of Hfq$_6$/Crc/*amiE*$_{\mathrm{6ARN}}$ RNA was mixed at equimolar concentrations and applied to a gel-filtration superdex S200 column equilibrated in a buffer containing 10 mM KCl, 40 mM NaCl, 1 mM Mg_2Cl, 20 mM HEPES pH 8.0, pure fractions were checked on 4-

12% SDS gels. The fractions containing Hfq, Crc and RNA were selected for the SEC-MAL experiment. 0.5 mg/ml BSA was used as a control.

Isolation of PAO1Δ*crcZ* revertants. The strains PAO1Δ*crcZ* (PAO6679) and PAO1Δ*crcZ*$_{ab}$ (PAO6713) containing either a promoter deletion or an entire deletion of the *crcZ* gene were pre-incubated in BSM medium supplemented with 40 mM succinate. The cells were washed in saline and approximately 10^8 cells were spread on plates containing 40 mM succinate, acetamide, mannitol, L-histidine and D/L-alanine, respectively. The plates were incubated at 37°C until single colonies appeared. The regions encompassing the *hfq* (corresponded to nt – 563 to nt +318 according to A (+1) of the start codon) and *crc* (corresponded to nt – 135 to nt + 971) genes of the revertants were amplified by PCR using primer pairs Q15 (5'-TTT TTT TTT TGG ATC CTC GGC GGG GTG TCG-3') and Q85 (5'-CCC TTC CAG ATG CAC CAG-3') for *hfq* and U3 (5'-GCT GGT GGT GAT CGG CTT C-3') and T3 (5'-GCA GAA CCC CGC GCT CG-3') for *crc*, respectively. The resulting PCR fragments were sequenced (barcode economy run, Microsynth) and compared with the wt sequence using Align Sequences Nucleotide BLAST (NCBI).

Qualitative determination of substrate utilization. Overnight cultures of PAO1, PAO1Δ*hfq*, PAO1Δ*crc* and PAO1Δ*crcZ* grown in BSM amended with 40 mM succinate were washed and diluted in 0.9% (wt/vol) NaCl to an OD_{600} of 0.045. 150 µl of the dilutions was used to inoculate each well of the GN2 MicroPlate™ (Biolog). The microplate was incubated at 35°C and rotated at 500 rpm in a THERMOstar+ apparatus (BMG Labtech). Each of the 96 wells of the plates contains a carbon source in addition to tetrazolium salts, which are reduced during respiration of the carbon source. This allows a growth independent visual screening of

the carbon sources that were respired. After 24 and 48 hours changes in colour were determined visually.

Determination of the intracellular Crc concentration. PAO1 was grown in BSM medium supplemented with 40 mM succinate to an OD_{600} of 2.0. The colony forming units (CFU)/ml were determined by plating serial dilutions on LB plates. To determine the Crc concentration three samples withdrawn from three individual cultures (corresponding to 50µl culture of PAO1 at an OD_{600} of 2.0). The samples were centrifuged and resuspended in protein loading buffer. Either sample was separated on a 12% SDS-polyacrylamide gel together with 0.5, 1 and 2 pmol of purified Crc protein. The proteins were then electro-blotted to a nitrocellulose membrane. The blot was blocked with 5% dry milk in TBS buffer, followed by probing with rabbit anti-Crc (Pineda) antibody. The antibody-antigen complexes were visualized with alkaline-phosphatase conjugated secondary antibodies (Sigma) using the chromogenic substrates nitro blue tetrazolium chloride (NBT) and 5-Bromo-4-chloro-3-indolyl phosphate (BCIP) and analysed by ImageQuant software. The Crc concentrations were determined with the aid of defined amounts of Crc protein loaded onto the gel. The Crc concentration(s) were then normalized to the CFU/ml and multiplied with the Avogadro constant resulting in the amount of Crc molecules/cell.

Supplementary References

59. Winsor, G.L., Griffiths, E.J., Lo, R., Dhillon, B.K., Shay, J.A., and Brinkman, F.S. (2016) Enhanced annotations and features for comparing thousands of *Pseudomonas* genomes in the *Pseudomonas* genome database. *Nucleic Acids Res*., **44**, D646-53.
60. Ye, R.W., Haas, D., Ka, J.O., Krishnapillai, V., Zimmermann, A., Baird, C., and Tiedje, J.M. (1995) Anaerobic activation of the entire denitrification pathway in *Pseudomonas aeruginosa* requires Anr, an analog of Fnr. *J. Bacteriol*., **177**, 3606-3609.
61. Holloway, B.W., Krishnapillai, V., and Morgan, A.F. (1979) Chromosomal genetics of *Pseudomonas*. *Microbiol. Rev*., **43**, 73-102.
62. Muffler, A., Fischer, D., and Hengge-Aronis, R. (1996) The RNA-binding protein HF-I, known as a host factor for phage Qbeta RNA replication, is essential for *rpoS* translation in *Escherichia coli*. *Genes Dev*., **10**, 1143-1151.
63. Steiner, M., Lubitz, W., and Bläsi, U. (1993) The missing link in phage lysis of gram-positive bacteria: gene 14 of Bacillus subtilis phage phi 29 encodes the functional homolog of lambda S protein. *J. Bacteriol*., **175**, 1038-1042.
64. Rist, M., and Kertesz, M.A. (1998) Construction of improved plasmid vectors for promoter characterization in *Pseudomonas aeruginosa* and other gram-negative bacteria. *FEMS Microbiol. Lett*., **169**, 179-183.
65. Karimova, G., Ullmann, A., and Ladant, D. (2001) Protein-protein interaction between *Bacillus stearothermophilus* tyrosyl-tRNA synthetase subdomains revealed by a bacterial two-hybrid system. *J. Mol. Microbiol. Biotechnol*., **3**, 73-82.
66. Claessen, D., Emmins, R., Hamoen, L.W., Daniel, R.A., Errington, J., and Edwards, D.H. (2008) Control of the cell elongation-division cycle by shuttling of PBP1 protein in *Bacillus subtilis*. *Mol. Microbiol*., **68**, 1029-1046.
67. Voisard, C., Bull, C.T., Keel, C., Laville, J., Maurhofer, M., Schnider, U., Défago, G., and Haas, D. (1994) Biocontrol of Root Diseases by *Pseudomonas fluorescens* CHA0: Current Concepts and Experimental Approaches. In O'Gara, F., Dowling, D., and Boesten, B., (eds.), *Molecular Ecology of Rhizosphere Microorganisms: Biotechnology and the release of GMOs*. Wiley-VCH Verlag GmbH, Weinheim, Germany, pp. 67–89.
68. Schuster, M., Lostroh, C.P., Ogi, T., and Greenberg, E.P. (2003) Identification, timing, and signal specificity of *Pseudomonas aeruginosa* quorum-controlled genes: a transcriptome analysis. *J. Bacteriol*., **185**, 2066-2079.
69. Lequette, Y., Lee, J.H., Ledgham, F., Lazdunski, A., and Greenberg, E.P. (2006) A distinct QscR regulon in the *Pseudomonas aeruginosa* quorum-sensing circuit. *J. Bacteriol*., **188**, 3365-3370.
70. Déziel, E., Gopalan, S., Tampakaki, A.P., Lépine, F., Padfield, K.E., Saucier, M., Xiao, G., and Rahme, L.G. (2005) The contribution of MvfR to *Pseudomonas aeruginosa* pathogenesis and quorum sensing circuitry regulation: multiple quorum sensing-regulated genes are modulated without affecting *lasRI*, *rhlRI* or the production of N-acyl-L-homoserine lactones. *Mol. Microbiol*., **55**, 998-1014.
71. Bredenbruch, F., Geffers, R., Nimtz, M., Buer, J., and Häussler, S. (2006) The *Pseudomonas aeruginosa* quinolone signal (PQS) has an iron-chelating activity. *Environ. Microbiol*., **8**, 1318-1329.
72. Wang, G., Huang, X., Li, S., Huang, J., Wei, X., Li, Y., and Xu, Y. (2012) The RNA chaperone Hfq regulates antibiotic biosynthesis in the rhizobacterium *Pseudomonas aeruginosa* M18. *J. Bacteriol*., **194**, 2443-2457.

Figure S1. Sonnleitner *et al*.

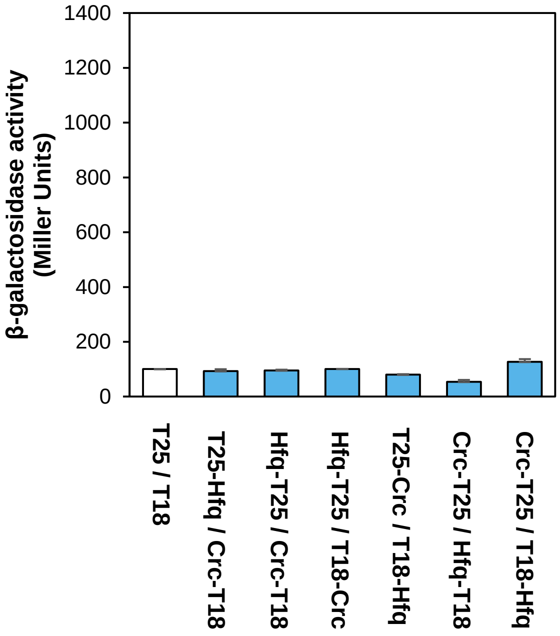

Figure S1. Combinations of Hfq and Crc fusion proteins with T18 and T25 of adenylate cyclase that did not restore adenylate cyclase activity. N-terminal and C-terminal fusion proteins of Crc and Hfq with T18 and T25 of adenylate cyclase were constructed as described in Text S1. The *E. coli* strain BTH101 was co-transformed with plasmids encoding the respective fusion proteins as indicated below the blue bars. Functional adenylate cyclase is only reconstituted when Crc and Hfq interact with each other, which is reflected by β-galactosidase production. White bar, background production of β-galactosidase in *E.coli* BTH101(pUT18, pKT25) harbouring the parental plasmids. The results of three independent experiments were averaged and are shown as mean ± standard deviation.

Figure S2. Sonnleitner *et al*.

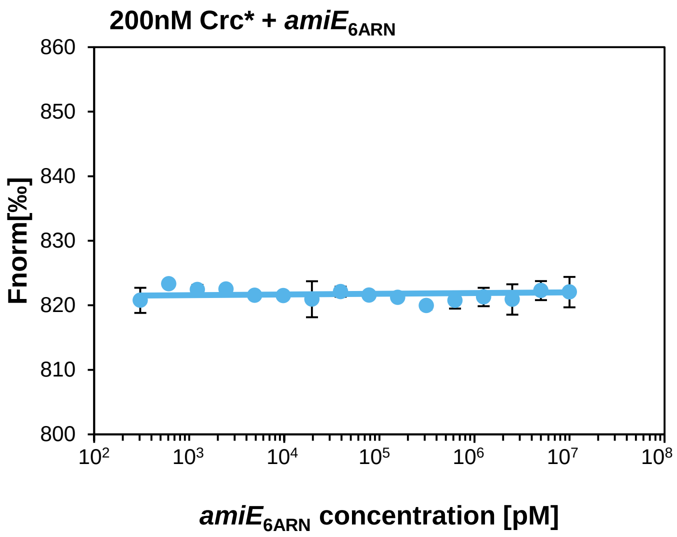

Figure S2. Crc does not interact with *amiE*$_{6ARN}$ RNA. MST analysis including 200 nM labelled Crc protein with increasing amounts of *amiE*$_{6ARN}$ RNA. Data from two independent experiments are shown as mean ± standard deviation. Thermophoresis/T-jump analysis is shown. LED power of 90% and MST power of 60% were used.

Figure S3. Sonnleitner *et al.*

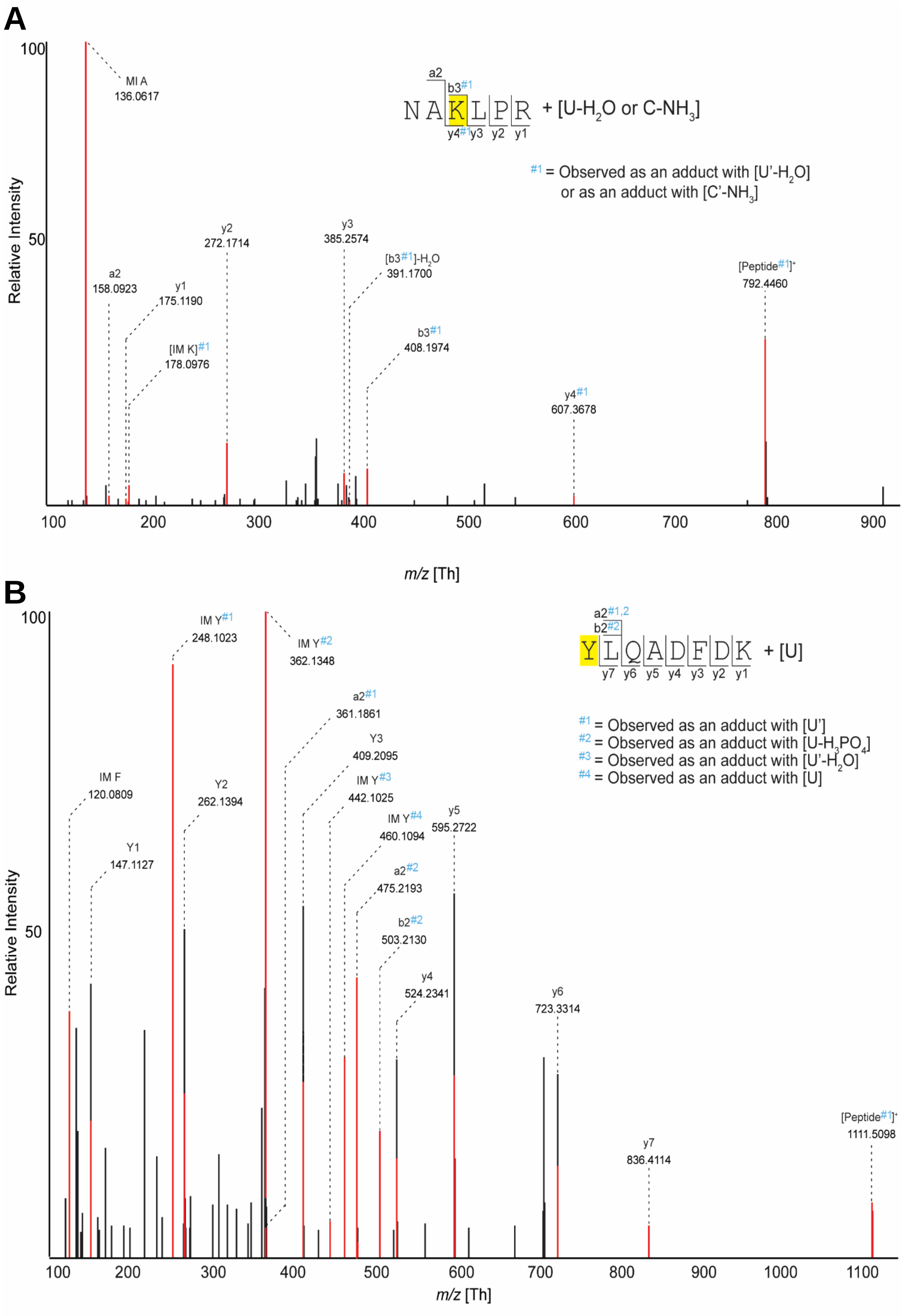

Figure S3. MS/MS spectra of RNA cross-linked Crc peptides identified in the Hfq/Crc/*amiE*$_{6ARN}$ complex. (**A**) The hexapeptide N_{234}-R_{239} cross-linked to either an uracil nucleotide or a cytosine nucleotide. The identification of both shifted y-ions and b-ions and one critical a-ion assigned the location of the cross-link to K_{236}, which is highlighted in yellow. (**B**) The octapeptide Y_{94}-K_{101} was found to be cross-linked to a uracil nucleotide, as identified by shifted a2-ions and b2-ions in addition to multiple differently shifted immonium ions. Thus, Y_{94} was identified to be the cross-linking amino acid. Ions with a mass shift of $\#^1$, $\#^2$, $\#^3$, and $\#^4$ correspond to the cross-linked nucleotides U', U-H_3PO_4, U'-H_2O and U, respectively. U: Uracil (324.04 Da); U': nucleobase of U (112.02 Da).

Figure S4. Sonnleitner *et al*.

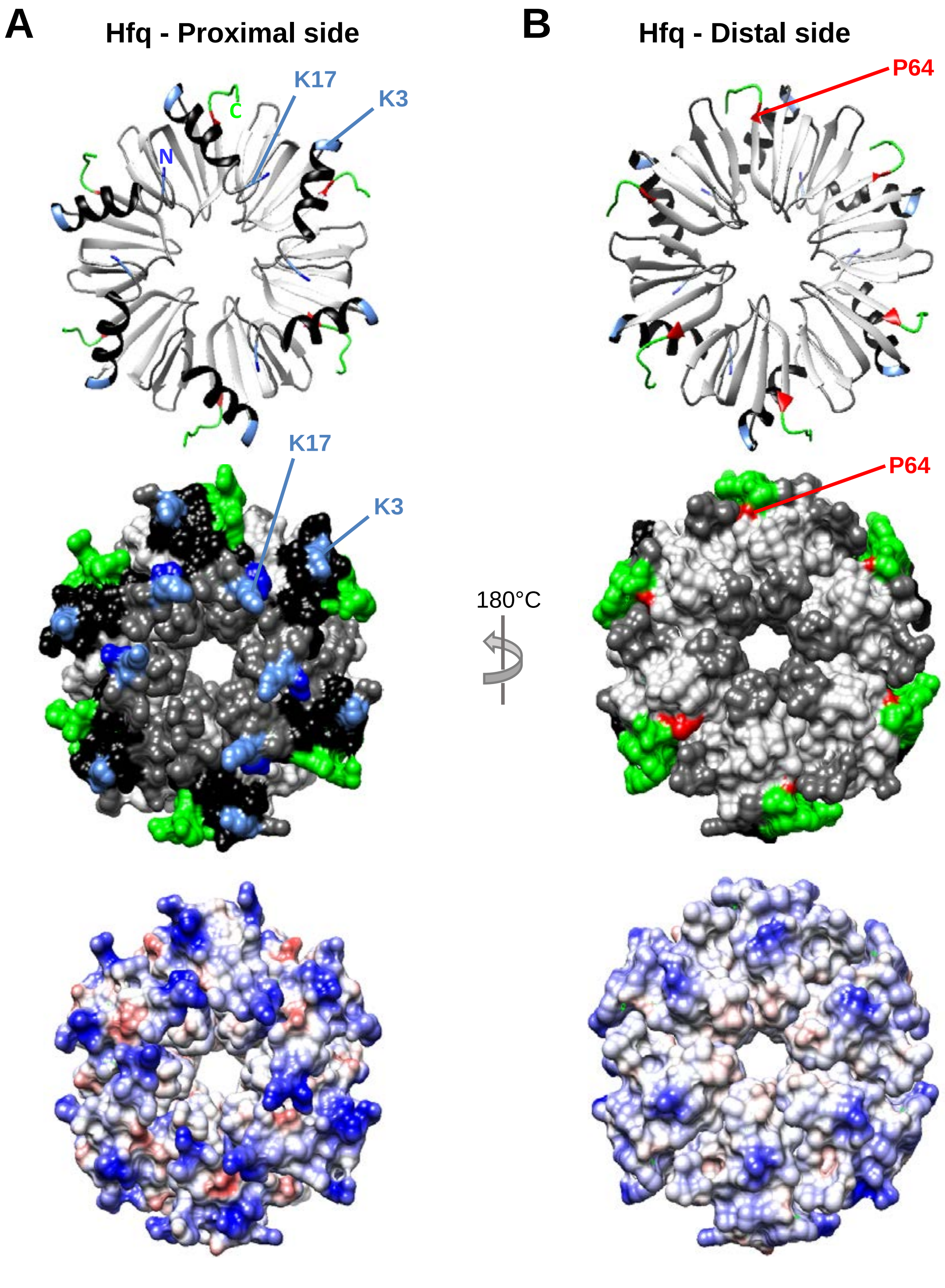

Figure S4. Location of K_3, K_{17} and P_{64} in Hfq. (**A-B**) Ribbon diagram (top), surface representation (middle) and electrostatic surface potential (bottom) of the proximal (**A**) and distal (**B**) side of Hfq (49). The Hfq structure lacks M_1 and amino acids 71-82 of the N- and C-terminus, respectively. Middle, the N- and C-termini are coloured in blue and green, respectively. K_3, K_{17} and P_{64} are highlighted in light blue and red, respectively. Image visualization was done with Chimera (38). The electrostatic surface potential (bottom) was calculated by Coulomb's law and visualized by Chimera (38). The electrostatic potential ranges from -10 (red) to +10 (blue) kcal/(mol**e*) at 298K.

Figure S5. Sonnleitner *et al*.

A **Hfq/Crc/RNA low salt**

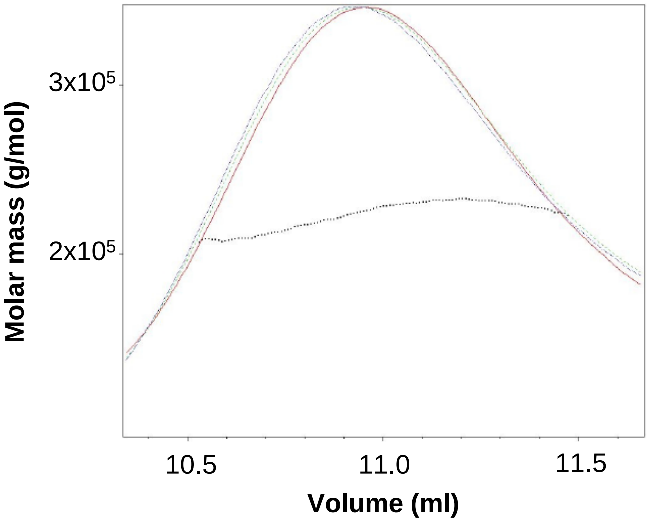

<u>Molar mass moments (g/mol)</u>
Mn: 2.196 x 10^5 (±0.047%)
Mw: 2.198 x 10^5 (±0.047%)
<u>Polydispersity</u>
Mw/Mn: 1.001 (±0.066%)

Observed MW Hfq/Crc/RNA: 220kDa

B **Hfq high salt**

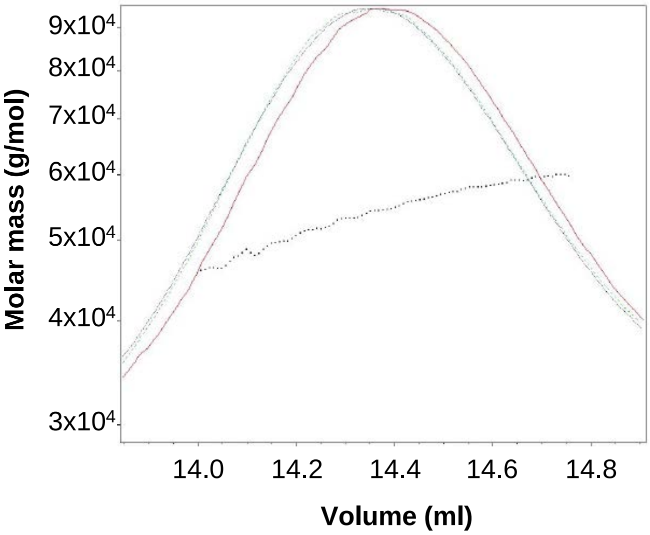

<u>Molar mass moments (g/mol)</u>
Mn: 5.315 x 10^4 (±0.356%)
Mw: 5.374 x 10^4 (±0.358%)
<u>Polydispersity</u>
Mw/Mn: 1.011 (±0.505%)

Observed MW Hfq: 53.7kDa
Predicted MW Hfq monomer: 9.1kDa
Hfq hexamer: 54.63kDa

C **Crc high salt**

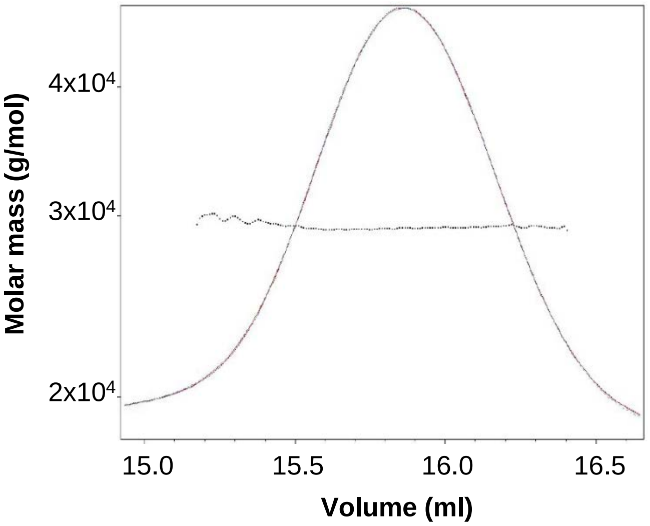

<u>Molar mass moments (g/mol)</u>
Mn: 2.959 x 10^4 (±0.494%)
Mw: 2.959 x 10^4 (±0.494%)
<u>Polydispersity</u>
Mw/Mn: 1.000 (±0.698%)

Observed MW Crc: 29.6kDa
Predicted MW Crc: 29.8kDa

Figure S5. SEC-MALS analysis of the Hfq/Crc/*amiE*$_{6ARN}$ complex. (**A**) SEC-MALS of the Hfq/Crc/*amiE*$_{6ARN}$ complex was performed in ES-buffer (10 mM Tris pH 8.0, 40 mM NaCl, 10 mM KCl and 1 mM $MgCl_2$). SEC-MALS of Hfq (**B**) and Crc (**C**) was performed in storage buffer (50 mM HEPES pH 8.0, 150 mM NaCl). Left: SEC-MALS elution profile showing estimated molecular mass variation over the elution profile (red line: LS light scattering intensity, green line: UV absorbance, blue line: refractive index change, black dotted line: molar mass). Right: summary of molecular mass estimates (Mn: number averaged, Mw: weight averaged) and polydispersity.

Figure S6. Sonnleitner *et al*.

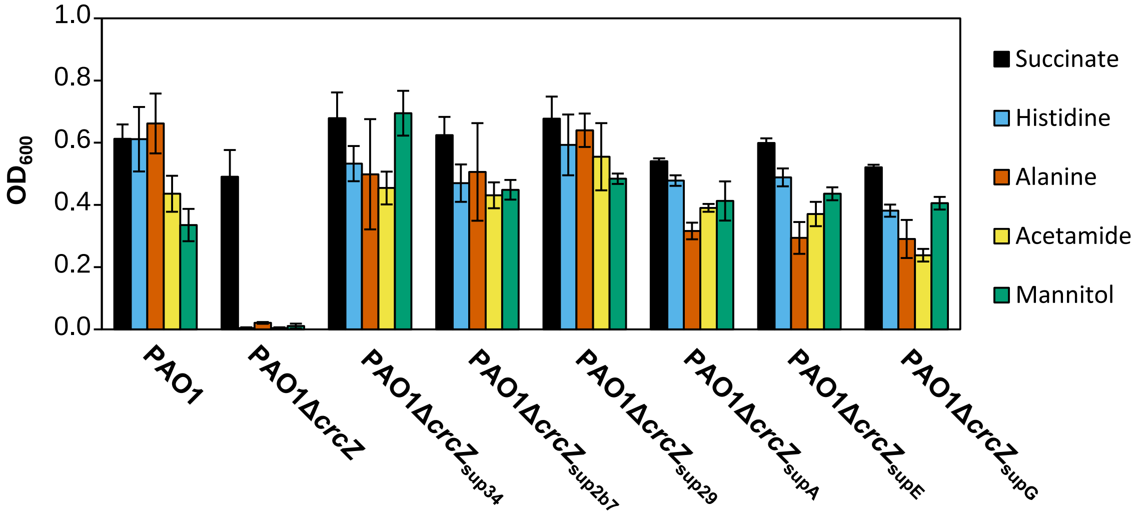

Figure S6. CCR is alleviated in PAO1Δ*crcZ* revertants. Single colonies of the strains PAO1, PAO1Δ*crcZ*, PAO1Δ$crcZ_{sup34}$, PAO1Δ$crcZ_{sup2b7}$, PAO1Δ$crcZ_{sup29}$, PAO1Δ$crcZ_{supA}$, PAO1Δ$crcZ_{supE}$ and PAO1Δ$crcZ_{supG}$, respectively, were inoculated in 100 µl of BSM medium supplemented with either 40 mM succinate, histidine, alanine, acetamide or mannitol (the colour code of the bars are shown at the right). The strains were incubated at 37°C under shaking. After 24 h the OD_{600} was measured with an iMark^{Tm} Microplate reader (Biorad). The results represent data from two independent experiments and are shown as mean ± standard deviation.

Figure S7. Sonnleitner *et al*.

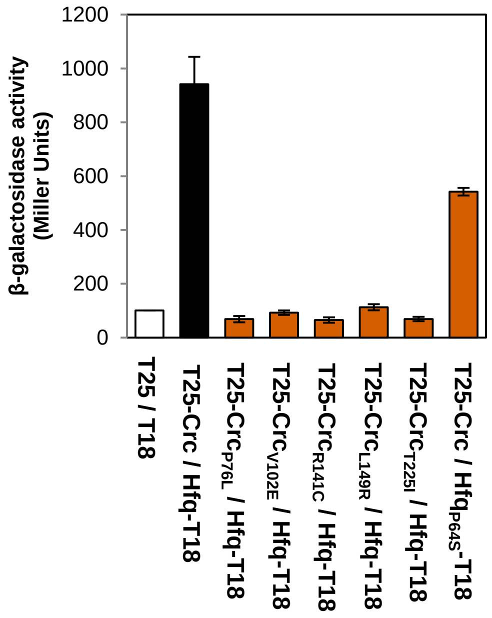

Figure S7. Hfq and Crc variants impaired in Hfq/Crc complex formation. (**A**) N-terminal and C-terminal fusion proteins of the Crc variants and the Hfq variant with T25 and T18 of adenylate cyclase, respectively, were constructed as described in Text S1. The *E. coli* strain BTH101 was co-transformed with plasmids encoding the respective fusion proteins as indicated below the orange bars. White bar, background production of β-galactosidase in *E. coli* BTH101(pUT18, pKT25) harbouring the parental plasmids. Black bar, co-synthesis of T25-Crc and Hfq-T18 results in reconstitution of the cyclase activity. The results of three independent experiments were averaged and are shown as mean ± standard deviation.

Figure S8. Sonnleitner *et al*.

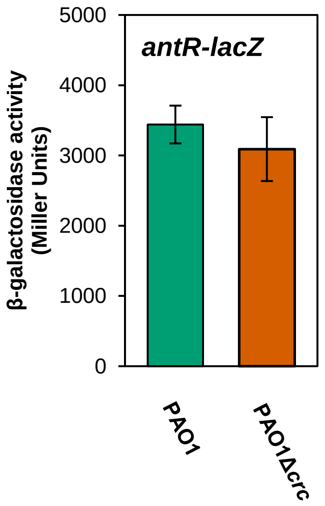

Figure S8. Crc does not impact on the promoter activity of *antR*. The strains PAO1 and PAO1Δ*crc* were grown to an OD_{600} of 2.0 in BSM medium supplemented with 40 mM succinate and 0.2 mM anthranilate. Anthranilate was added to induce *antR* transcription. The bars depict the β-galactosidase activity conferred by the transcriptional *antR*-*lacZ* fusion encoded by plasmid pTCantR in strain PAO1 (green bar) and PAO1Δ*crc* (orange bar).

Figure S9. Sonnleitner *et al.*

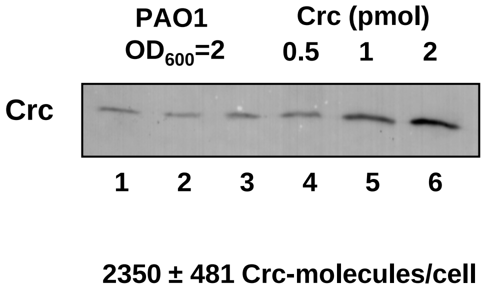

Figure S9. Determination of the intracellular Crc concentration. Strain PAO1 was grown to an OD_{600} of 2.0 in BSM medium supplemented with 40 mM succinate (= 3.2 ± 0.3 10^9 CFU/ml). The Crc concentration was determined in triplicate samples of PAO1 cell lysates corresponding to 50 µl of culture (lanes 1–3) using quantitative western-blotting with Crc specific antibodies. Lanes 4-6, 0.5, 1 and 2 pmol of purified Crc protein were loaded, respectively. The Crc concentration per cell was determined as described in Text S1.

Table S1. Strains and plasmids used in this study

Strain/plasmid	Genotype/relevant features	Source/reference
P. aeruginosa		
PAO1		(61)
PAO1*hfq-*	*hfq::aadA*; Sp/Sm^r	(10)
PAO1Δ*hfq*	In frame deletion of *hfq* from base pair 5548400 to 5548645 (59)	(14)
PAOΔ*crc*	PAO6673, *crc* deletion from base pair 6002121 to 6002899 (59)	(8)
PAO1Δ*crcZ*	PAO6679, *crcZ* promoter deletion from base pair 5308523 to 5308682 (59)	(8)
PAO1Δ*crc*Δ*crcZ*	PAO6673 with a deletion in the *crcZ* promoter encompassing base pairs 5308523 to 5308682 (59)	This study
PAO1Δ$crcZ_{sup29}$	PAO6679, Crc_{L149R}	This study
PAO1Δ$crcZ_{sup2b7}$	PAO6679, Crc_{V102E}	This study
PAO1Δ$crcZ_{sup34}$	PAO6679, Hfq_{P64S}	This study
PAO1Δ$crcZ_{ab}$	PAO6713, deletion of the *crcZ* gene from base pair 5308523 to 5308930 (59)	This study
PAO1Δ$crcZ_{supA}$	PAO6713, Crc_{P76L}	This study
PAO1Δ$crcZ_{supE}$	PAO6713, Crc_{T225I}	This study
PAO1Δ$crcZ_{supG}$	PAO6713, Crc_{R141C}	This study
E. coli		
BTH101	*F$^-$, cya-99, araD139, galE15, galK16, rpsL1 (Str^r), hsdR2, mcrA1, mcrB1*	(31)
BL21(DE3)	*F$^-$, ompT, hsdS$_B$(r$_B$-, m$_B$-), dcm, gal, λ*(DE3)	Novagen
AM111F'	AM111 *hfq::Km^r* [*F' proAB lacI^q lacZ*ΔM15::Tn10; Tc^r]	(62)
MC4100F′	*lac I^Q araDU169* Sm^r *thi relA* [F' *proAB lacl lacZΔM15*::Tn10; Tc^r]	(63)
**Plasmids**		
pME4510	Broad–host-range promoter-probe plasmid, Gm^r	(64)
pME4510hfq_{Flag}	pME4510 carrying PAO1 *hfq* fused to a Flag-tag encoding sequence under control of its authentic promoter	(7)
pME4510crc_{Flag}	pME4510 carrying PAO1 *crc* fused to a Flag-tag encoding sequence under control of its authentic promoter	This study
pUT18	P_{lac}-mcs-$cyaA_{673\text{-}1197}$, Ap^r (contains T18 domain)	(65)
pUT18C	P_{lac}-$cyaA_{673\text{-}1197}$-mcs, Ap^r (contains T18 domain)	(65)
pKT25	P_{lac}-$cyaA_{1\text{-}672}$-mcs, Kan^r (contains T25 domain)	(65)
p25-N	P_{lac}-mcs-$cyaA_{1\text{-}672}$, Kan^r (contains T25 domain)	(66)
pKT25*hfq*	pKT25 encoding a T25 – PAO1-Hfq fusion	This study
p25-N*hfq*	p25-N encoding a PAO1-Hfq – T25 fusion	This study
pUT18*hfq*	pUT18 encoding a PAO1-Hfq – T18 fusion	This study
pUT18C*hfq*	pUT18C encoding a T18 – PAO1-Hfq fusion	This study
pKT25*crc*	pKT25 encoding a T25 – PAO1-Crc fusion	This study
p25-N*crc*	p25-N encoding a PAO1-Crc – T25 fusion	This study
pUT18*crc*	pUT18 encoding a PAO1-Crc – T18 fusion	This study
pUT18C*crc*	pUT18C encoding a T18 – PAO1-Crc fusion	This study
pUT18hfq_{Ec}	pUT18 encoding an *E. coli* Hfq – T18 fusion	This study
pKT25crc_{P76L}	pKT25crc encoding the PAO1 Crc_{P76L} mutant protein	This study
pKT25crc_{V102E}	pKT25crc encoding the PAO1 Crc_{V102E} mutant protein	This study
pKT25crc_{R141C}	pKT25crc encoding the PAO1 Crc_{R141C} mutant protein	This study
pKT25crc_{L149R}	pKT25crc encoding the PAO1 Crc_{L149R} mutant protein	This study
pKT25crc_{T225I}	pKT25crc encoding the PAO1 Crc_{T225I} mutant protein	This study
pUT18hfq_{P64S}	pUT18hfq encoding the PAO1 Hfq_{P64S} mutant protein	This study
pUT18hfq_{Y25D}	pUT18hfq encoding the PAO1 Hfq_{Y25D} mutant protein	This study
pET22bII-Crc	Overexpression plasmid carrying a C-terminal cleavable His_6-tagged *crc* gene under the control of a T7 promoter with a C-terminal thrombin cleavage site, Km^r	This study
pETM14lic-His_6Crc	Overexpression plasmid carrying a N-terminal cleavable His_6-tagged *crc* gene under the control of a T7 promoter	(24)
$pHfq_{Pae}$	pUC19 derivative harbouring PAO1 *hfq* under transcriptional control of P_{lac} and translational control of the T7 gene *10* rbs.	(7)
pME9655	carrying a translational *amiE::lacZ* fusion; Tc^r	(8)
pTLantR	carrying a translational *antR::lacZ* fusion; Tc^r	(14)
pTCantR	carrying a transcriptional *antR-lacZ* fusion; Tc^r	(14)
pME3087	Suicide vector, ColE1 replicon, Mob; Tc^r	(67)
pME9679	pME3087 with a 408-bp deletion of *crcZ*	This study
pME9673	pME3087 with a 779-bp deletion of *crc*	(8)

Table S2. Non-overlapping transcripts with decreased and increased abundance in PAO1*hfq*- versus PAO1

ORF[a]	Gene	Function[a]	Fold change *hfq*- vs wt (p-value)
PA4552	*pilW*	type 4 fimbrial biogenesis protein PilW	-19.6 (7.6E-42)
PA1869		probable acyl carrier protein	-18.5 (3.8E-72)
PA4553	*pilX*	type 4 fimbrial biogenesis protein PilX	-17.1 (3.7E-48)
PA4550	*fimU*	type 4 fimbrial biogenesis protein FimU	-17.0 (1.3E-48)
PA3361	*lecB*	fucose-binding lectin PA-IIL	-16.4 (2.9E-30)
PA4551	*pilV*	type 4 fimbrial biogenesis protein PilV	-16.2 (2.1E-43)
PA0171		hypothetical protein	-14.4 (1.8E-06)
PA4555	*pilY2*	type 4 fimbrial biogenesis protein PilY2	-14.1 (3.1E-62)
PA4556	*pilE*	type 4 fimbrial biogenesis protein PilE	-13.6 (3.1E-50)
PA4943		probable GTP-binding protein	-11.5 (4.9E-60)
PA1123		hypothetical protein	-10.3 (6.4E-46)
PA4208	*opmD*	probable outer membrane efflux protein precursor	-10.0 (1.3E-18)
PA5024		conserved hypothetical protein	-9.9 (1.6E-12)
PA5267	*hcpB*	secreted protein Hcp	-9.6 (1.5 E-07)
PA1864		probable transcriptional regulator	-9.4 (4.7E-14)
PA0172	*siaA*	putative membrane protein with a HAMP and a PP2C-like phosphatase domain	-9.0 (6.3E-07)
PA0169	*siaD*	SiaD c-di-GMP synthetase	-8.9 (1.1E-11)
PA3326	*clpP2*	ClpP2 protease	-8.9 (2.3E-44)
PA3479	*rhlA*	rhamnosyltransferase chain A	-7.2 (5.4E-32)
PA1662	*clpV2*	protein secretion by the type VI secretion system	-7.1 (1.9E-25)
PA1639		hypothetical protein	-6.9 (2.6E-30)
PA1661	*hsiH2*	predicted component of the type VI protein secretion system	-6.9 (1.0E-15)
PA1812	*mltD*	membrane-bound lytic murein transglycosylase D precursor	-6.8 (2.8E-37)

PA4517		conserved hypothetical protein	-6.8 (4.3E-02)
PA4207	*mexI*	probable RND efflux transporter	-6.7 (6.5E-25)
PA1663	*sfa2*	Sfa2 protein secretion by the type VI secretion system	-6.6 (6.5E-13)
PA1668	*dotU2*	DotU2 type VI protein secretion system component	-6.5 (4.1E-17)
PA4554	*pilY1*	type 4 fimbrial biogenesis protein PilY1	-6.5 (2.8E-37)
PA1660	*hsiG2*	HsiG2 type VI protein secretion system component	-6.4 (8.7E-17)
PA3486		conserved hypothetical protein	-6.4 (2.0E-08)
PA1667	*hsiJ2*	HsiJ2 component of the type VI protein secretion system	-6.2 (6.5E-14)
PA2780		hypothetical protein	-6.1 (5.6E-15)
PA0758		hypothetical protein	-6.0 (4.3E-25)
PA2698		probable hydrolase	-5.5 (1.7E-21)
PA4031	*ppa*	inorganic pyrophosphatase	-5.5 (4.0E-24)
PA0284		hypothetical protein	-5.4 (2.3E-27)
PA1828		probable short-chain dehydrogenase	-5.3 (2.9E-23)
PA2204		probable binding protein component of ABC transporter	-5.3 (9.5E-31)
PA3397	*fpr*	NADP+-dependent ferredoxin reductase	-5.3 (2.0E-19)
PA0122	*rahU*	RahU protein	-5.2 (4.1E-10)
PA3450	*lsfA*	1-Cys peroxiredoxin LsfA	-5.2 (1.3E-08)
PA1669	*icmF2*	IcmF2 type VI protein secretion system component	-5.1 (8.0E-11)
PA5509		hypothetical protein	-5.1 (6.5E-21)
PA2264		conserved hypothetical protein	5.0 (1.0E-26)
PA1210		conserved hypothetical protein	5.0 (1.1E-20)
PA1105	*fliJ*	flagellar protein FliJ	5.0 (3.6E-26)
PA5452	*wbpW*	phosphomannose isomerase/GDP-mannose WbpW	5.1 (1.5E-26)
PA1052		conserved hypothetical protein	5.2 (1.3E-23)
PA4770	*lldP*	L-lactate permease	5.3 (1.8E-29)

PA5436		probable biotin carboxylase subunit of a transcarboxylase	5.4 (5.6E-31)
PA1025	*opdD*	probable porin	5.5 (1.4E-20)
PA0887	*acsA*	acetyl-coenzyme A synthetase	5.5 (4.8E-30)
PA1070	*braG*	branched-chain amino acid transport protein BraG	5.5 (1.2E-27)
PA2013	*liuC*	putative 3-methylglutaconyl-CoA hydratase	5.5 (9.1E-06)
PA0565		conserved hypothetical protein	5.5 (8.9E-14)
PA0106	*coxA*	cytochrome c oxidase. subunit I	5.7 (6.0E-27)
PA1693	*pscR*	translocation protein in type III secretion	5.7 (4.5E-07)
PA5351	*rubA1*	rubredoxin	5.7 (2.5E-30)
PA0112		hypothetical protein	5.7 (7.1E-20)
PA0107		conserved hypothetical protein	5.7 (1.0E-22)
PA1071	*braF*	branched-chain amino acid transport protein BraF	5.7 (1.9E-29)
PA2868		hypothetical protein	5.8 (4.9E-12)
PA1338	*ggt*	gamma-glutamyltranspeptidase precursor	5.9 (1.2E-31)
PA1418		probable sodium:solute symport protein	5.9 (3.9E-19)
PA2511	*antR*	transcriptional regulator AntR	5.9 (7.6E-33)
PA0247	*pobA*	p-hydroxybenzoate hydroxylase	6.0 (7.3E-18)
PA3583	*glpR*	glycerol-3-phosphate regulon repressor	6.1 (5.2E-28)
PA1485		probable amino acid permease	6.1 (2.0E-18)
PA3192	*gltR*	two-component response regulator GltR	6.2 (4.6E-34)
PA0321		probable acetylpolyamine aminohydrolase	6.2 (1.2E-19)
PA5545		conserved hypothetical protein	6.3 (2.0E-34)
PA4500		probable binding protein component of ABC transporter	6.3 (2.3E-33)
PA2002		conserved hypothetical protein	6.4 (1.3E-27)
NC		hypothetical protein	6.4 (3.0E-27)
PA5325	*sphA*	SphA protein	6.4 (7.1E-12)

PA3181	*edaA*	2-keto-3-deoxy-6-phosphogluconate aldolase	6.6 (6.1E-37)
PA0242		hypothetical protein	6.6 (8.7E-27)
PA1072	*braE*	branched-chain amino acid transport protein BraE	6.8 (1.8E-35)
PA3892		conserved hypothetical protein	6.9 (2.6E-28)
PA5154		probable permease of ABC transporter	6.9 (1.6E-29)
PA2321		gluconokinase	7.0 (2.4E-33)
PA3185		hypothetical protein	7.0 (9.3E-39)
PA3191	*gtrS*	glucose transport sensor. GtrS	7.0 (1.2E-37)
PA2188		probable alcohol dehydrogenase	7.1 (2.9E-03)
PA1991		probable iron-containing alcohol dehydrogenase	7.1 (9.0E-32)
PA1761		hypothetical protein	7.1 (4.4E-36)
PA1985	*pqqA*	pyrroloquinoline quinone biosynthesis protein A	7.1 (4.2E-27)
PA2014	*liuB*	methylcrotonyl-CoA carboxylase. beta-subunit	7.3 (7.4E-08)
PA4520		probable chemotaxis transducer	7.3 (5.4E-40)
PA1999	*dchA*	DchA. dehydrocarnitine CoA transferase. subunit A	7.3 (2.5E-05)
PA4596		probable transcriptional regulator	7.5 (3.2E-15)
PA0783	*putP*	sodium/proline symporter PutP	7.5 (2.8E-09)
PA0322		probable transporter	7.6 (9.6E-25)
PA1764		hypothetical protein	7.9 (1.9E-33)
PA3195	*gapA*	glyceraldehyde 3-phosphate dehydrogenase	8.0 (2.1E-43)
PA2379		probable oxidoreductase	8.1 (4.6E-35)
PA4973	*thiC*	thiamin biosynthesis protein ThiC	8.2 (2.5E-45)
PA1073	*braD*	branched-chain amino acid transport protein BraD	8.3 (1.3E-41)
PA1710	*exsC*	ExsC. exoenzyme S synthesis protein C precursor	8.3 (1.5E-03)
PA1948	*rbsC*	membrane protein component of ABC ribose transporter	8.5 (3.5E-42)
PA0119		probable dicarboxylate transporter	8.5 (5.2E-38)

PA5348		probable DNA-binding protein	8.7 (1.8E-46)
PA3535		probable serine protease	8.7 (1.2E-40)
PA3893		conserved hypothetical protein	8.9 (6.5E-43)
PA3569	*mmsB*	3-hydroxyisobutyrate dehydrogenase	9.1 (1.5E-29)
PA1019	*mucK*	cis.cis-muconate transporter MucK	9.2 (1.2E-28)
PA0228	*pcaF*	beta-ketoadipyl CoA thiolase PcaF	9.4 (2.1E-16)
PA1542		hypothetical protein	9.5 (5.8E-11)
PA5542		hypothetical protein	9.9 (2.2E-44)
PA1711	*exsE*	ExsE.	10.0 (1.2E-03)
PA1763		hypothetical protein	10.2 (1.7E-44)
PA2378		probable aldehyde dehydrogenase	10.3 (1.5E-35)
PA2552		probable acyl-CoA dehydrogenase	10.3 (1.2E-03)
PA1713	*exsA*	transcriptional regulator ExsA	10.6 (6.1E-04)
PA3182	*pgl*	6-phosphogluconolactonase	11.2 (3.0E-56)
PA4909		probable ATP-binding component of ABC transporter	11.2 (5.6E-18)
PA3183	*zwf*	glucose-6-phosphate 1-dehydrogenase	11.3 (2.0E-57)
PA5543		hypothetical protein	11.6 (5.6E-37)
PA4985		hypothetical protein	12.2 (2.6E-41)
PA2553		probable acyl-CoA thiolase	12.3 (3.4E-04)
PA1712	*exsB*	exoenzyme S synthesis protein B	12.6 (3.2E-04)
PA2554		probable short-chain dehydrogenase	13.1 (7.1E-05)
PA3519		hypothetical protein	13.2 (2.9E-03)
PA5544		conserved hypothetical protein	13.6 (9.1E-58)
PA2515	*xylL*	cis-1.2-dihydroxycyclohexa-3.4-diene carboxylate dehydrogenase	13.7 (1.9E-09)
PA4153		2.3-butanediol dehydrogenase	13.7 (5.8E-13)
PA3516		probable lyase	13.9 (3.4E-06)

PA4913		probable binding protein component of ABC transporter	13.9 (5.2E-20)
PA5380	*gbdR*	glycine betaine- and dimethylglycine-responsive regulator	14.1 (3.0E-55)
PA4093		hypothetical protein	14.3 (5.8E-42)
PA3517		probable lyase	14.4 (5.1E-04)
PA0531		probable glutamine amidotransferase	14.4 (1.1E-17)
PA4908		hypothetical protein	14.5 (9.5E-19)
PA1988	*pqqD*	pyrroloquinoline quinone biosynthesis protein D	14.7 (6.8E-21)
PA1692	*pscS*	probable translocation protein in type III secretion	15.0 (1.5E-06)
PA1989	*pqqE*	pyrroloquinoline quinone biosynthesis protein E	15.5 (7.1E-17)
PA0530		probable class III pyridoxal phosphate-dependent aminotransferase	15.8 (1.5E-19)
PA1987	*pqqC*	pyrroloquinoline quinone biosynthesis protein C	15.9 (1.8E-12)
PA1990	*pqqH*	PqqH	16.6 (3.3E-33)
PA1992	*ercS*	phosphorelay sensor kinase activity	17.5 (1.2E-33)
PA3360		probable secretion protein	18.0 (9.0E-68)
PA3709		probable MFS transporter	18.3 (1.2E-63)
PA2682		conserved hypothetical protein	19.2 (2.8E-78)
PA1714	*exsD*	ExsD. negative regulator of type III secretion regulon	19.3 (4.3E-04)
PA4150		probable dehydrogenase E1 component	20.3 (6.1E-22)
PA3233		hypothetical protein	20.5 (9.3E-26)
PA1986	*pqqB*	pyrroloquinoline quinone biosynthesis protein B	20.5 (2.9E-13)
PA3568		probable acetyl-coa synthetase	20.5 (2.6E-72)
PA3235		conserved hypothetical protein	20.8 (1.5E-08)
PA2505	*opdT*	tyrosine porin OpdT	20.8 (5.6E-32)
PA0227		probable CoA transferase. subunit B	21.1 (2.8E-16)
PA1690	*pscU*	translocation protein in type III secretion	22.0 (1.3E-07)
PA4025		probable ethanolamine ammonia-lyase light chain	22.2 (1.3E-33)

PA4501	*opdP*	Glycine-glutamate dipeptide porin OpdP	22.6 (1.6E-71)
PA4910		branched chain amino acid ABC transporter ATP binding protein	22.7 (3.9E-17)
PA1704	*pcrR*	transcriptional regulator protein PcrR	23.1 (1.3E-08)
PA0226		probable CoA transferase. subunit A	23.9 (9.3E-42)
PA1691	*pscT*	translocation protein in type III secretion	24.9 (5.2E-08)
PA4152		probable hydrolase	24.9 (5.3E-16)
PA4148		probable short-chain dehydrogenase	26.3 (9.9E-09)
PA1898	*qscR*	quorum-sensing control repressor	27.2 (1.5E-16)
PA4149		conserved hypothetical protein	29.7 (2.7E-17)
PA0235	*pcaK*	4-hydroxybenzoate transporter PcaK	31.7 (1.7E-63)
PA1703	*pcrD*	type III secretory apparatus protein PcrD	31.9 (1.9E-08)
PA4024	*eutB*	ethanolamine ammonia-lyase large subunit	32.8 (2.4E-68)
PA1718	*pscE*	type III export protein PscE	34.8 (7.4E-08)
PA1706	*pcrV*	type III secretion protein PcrV	36.3 (2.3E-04)
PA1725	*pscL*	type III export protein PscL	36.7 (2.7E-06)
PA1700	*pcr2*	Pcr2 conserved hypothetical protein in type III secretion	36.8 (3.0E-06)
PA1723	*pscJ*	type III export protein PscJ	38.3 (2.3E-08)
PA2250	*lpdV*	lipoamide dehydrogenase-Val	38.9 (6.9E-108)
PA1699	*pcr1*	Pcr1. negative regulation of protein secretion	39.8 (4.0E-06)
PA1705	*pcrG*	regulator in type III secretion	40.0 (1.0E-04)
PA3038		probable porin	41.0 (3.2E-18)
PA1709	*popD*	Translocator outer membrane protein PopD precursor	41.3 (3.3E-05)
PA1722	*pscI*	type III export protein PscI	41.7 (3.2E-08)
PA1721	*pscH*	type III export protein PscH	41.8 (1.3E-08)
PA1720	*pscG*	type III export protein PscG	43.3 (6.5E-08)
PA1715	*pscB*	type III export apparatus protein	43.6 (5.3E-06)

PA1719	*pscF*	type III export protein PscF	43.7 (8.7E-09)
PA4911		probable permease of ABC branched-chain amino acid transporter	45.7 (5.7E-33)
PA1707	*pcrH*	regulatory protein PcrH	46.0 (7.2E-05)
PA1698	*popN*	Type III secretion outer membrane protein PopN precursor	46.3 (1.9E-05)
PA1717	*pscD*	type III export protein PscD	47.3 (3.2E-07)
PA1694	*pscQ*	translocation protein in type III secretion	48.3 (9.9E-11)
PA1696	*pscO*	translocation protein in type III secretion	48.8 (2.1E-09)
PA4912		branched chain amino acid ABC transporter membrane protein	51.6 (3.2E-57)
PA1697	*pscN*	ATP synthase in type III secretion system	51.7 (1.1E-07)
PA1695	*pscP*	translocation protein in type III secretion	51.8 (3.9E-15)
PA1716	*pscC*	Type III secretion outer membrane protein PscC precursor	53.8 (3.9E-07)
PA1708	*popB*	translocator protein PopB	54.6 (2.0E-05)
PA1724	*pscK*	type III export protein PscK	54.6 (2.7E-07)
PA3843		hypothetical protein	55.8 (1.8E-04)
PA3842	*spcS*	specific *Pseudomonas* chaperone for ExoS. SpcS	56.7 (7.2E-05)
PA2191	*exoY*	adenylate cyclase ExoY	56.9 (7.6E-05)
PA1702	*pcr4*	Pcr4 protein secretion by the type III secretion system	57.6 (3.0E-07)
PA3518		hypothetical protein	59.3 (4.7E-08)
PA3841	*exoS*	exoenzyme S	59.6 (1.7E-03)
PA4151	*acoB*	acetoin catabolism protein AcoB	59.6 (6.4E-29)
PA2249	*bkdB*	branched-chain alpha-keto acid dehydrogenase	61.2 (4.6E-128)
PA3234		probable sodium:solute symporter	61.9 (1.6E-15)
PA1975		hypothetical protein	64.7 (1.2E-42)
PA1892		hypothetical protein	66.1 (6.3E-67)
PA1976	*ercS'*	phosphorelay sensor kinase activity	66.6 (3.9E-46)
PA2508	*catC*	muconolactone delta-isomerase	67.0 (7.7E-97)

PA1701	*pcr3*	Pcr3 protein secretion by the type III secretion system	70.6 (7.8E-08)
PA0044	*exoT*	exoenzyme T	70.9 (2.3E-04)
PA1891		hypothetical protein	71.7 (7.8E-66)
PA2247	*bkdA1*	2-oxoisovalerate dehydrogenase (alpha subunit)	77.6 (6.5E-139)
PA2248	*bkdA2*	2-oxoisovalerate dehydrogenase (beta subunit)	85.7 (9.0E-143)
PA2322		gluconate permease	89.4 (1.4E-135)
PA1894		hypothetical protein	94.9 (2.0E-65)
PA2509	*catB*	muconate cycloisomerase I	96.0(6.1E-143)
PA1893		hypothetical protein	103.9 (5.3E-69)
PA1974		hypothetical protein	109.2 (2.7E-11)
PA1895		hypothetical protein	112.3 (5.3E-83)
PA1980	*eraR*	response regulator EraR	129.4 (1.4E-139)
PA2507	*catA*	catechol 1.2-dioxygenase	138.0 (1.1E-83)
PA1981		hypothetical protein	139.7 (6.5E-21)
PA1979	*eraS*	sensor kinase EraS	170.0 (1.2E-142)
PA1896		hypothetical protein	187.3 (4.0E-62)
PA1978	*erbR*	response regulator ErbR	212.5 (6.2E-108)
PA1982	*exaA*	quinoprotein alcohol dehydrogenase	268.5 (9.6E-25)
PA1897		hypothetical protein	358.5 (4.4E-68)
PA1977		hypothetical protein	435.9 (8.5E-179)
PA1984	*exaC*	NAD+ dependent aldehyde dehydrogenase ExaC	479.0 (5.2E-30)
PA1983	*exaB*	cytochrome c550	518.8 (1.3E-28)

[a] Gene numbers and functions are taken from the *Pseudomonas* genome database (59).

Table S3. Non-overlapping transcripts with decreased and increased abundance in PAO1Δ*crc* versus PAO1

**ORF**[a]	**Gene**	**Function**[a]	**Fold change Δ*crc* *vs* wt (p-value)**
PA4739		conserved hypothetical protein	-23.1 (1.1E-30)
PA4738		conserved hypothetical protein	-23.0 (4.0E-16)
PA5482		hypothetical protein	-21.5 (5.6E-97)
PA5481		hypothetical protein	-16.9 (9.2E-97)
PA2433		hypothetical protein	-11.4 (4.3E-10)
PA1404		hypothetical protein	-10.5 (2.6E-05)
PA2146		conserved hypothetical protein	-10.0 (3.8E-09)
PA3692	*lptF*	Lipotoxon F	-9.7(4.7E-07)
PA0355	*pfpI*	protease PfpI	-9.4 (2.2E-04)
PA3691		hypothetical protein	-9.2 (1.7E-05)
PA4880		putative bacterioferritin	-8.8 (9.4E-04)
PA1246	*aprD*	alkaline protease secretion protein AprD	-8.6 (7.1E-32)
PA0059	*osmC*	osmotically inducible protein OsmC	-8.3 (1.9E-04)
PA2171		hypothetical protein	-8.2 (3.3E-06)
PA1245	*aprX*	Type I secretion protein AprX	-8.0 (2.1E-65)
PA4306	*flp*	Type IVb pilin, Flp	-7.7 (9.0E-02)
PA4876	*osmE*	osmotically inducible lipoprotein OsmE	-7.4 (2.2E-06)
PA0567		conserved hypothetical protein	-7.2 (5.5E-05)
PA1137		probable oxidoreductase	-6.91.2E-10)
PA1323		hypothetical protein	-6.8 (2.5E-05)
PA1324		hypothetical protein	-6.7 (8.8E-05)

PA2176		hypothetical protein	-6.2 (1.5E-19)
PA2747		hypothetical protein	-6.2 (5.1E-25)
PA1216		hypothetical protein	-6.1 (4.7E-25)
PA4290		probable chemotaxis transducer	-6.0 (4.8E-08)
PA5100	*hutU*	urocanase	-6.0 (3.7E-04)
PA2166		hypothetical protein	-5.8 (8.3E-36)
PA0958	*oprD*	Basic amino acid, basic peptide and imipenem outer membrane porin OprD precursor	-5.4 (3.7E-46)
PA4811	*fdnH*	nitrate-inducible formate dehydrogenase, beta subunit	-5.1 (8.2E-04)
PA0434		hypothetical protein	5.3 (6.5E-06)
PA3598		conserved hypothetical protein	5.4 (2.1E-17)
PA4838		hypothetical protein	5.7 (3.8E-18)
PA3430		probable aldolase	6.0 (1.4E-47)
PA2913		hypothetical protein	7.2 (8.9E-15)
PA3597		probable amino acid permease	7.4 (1.6E-41)
PA3432		hypothetical protein	7.5 (2.0E-11)
PA2914		probable permease of ABC transporter	8.2 (4.4E-19)
PA2912		probable ATP-binding component of ABC transporter	8.2 (5.4E-23)
PA2911		probable TonB-dependent receptor	8.3 (2.9E-19)
PA3431		conserved hypothetical protein	9.5 (1.9E-35)
PA0754		hypothetical protein	23.2 (1.1E-40)
PA0752		hypothetical protein	25.3 (3.0E-37)
PA0751		conserved hypothetical protein	28.0 (6.2E-119)
PA0753		hypothetical protein	32.4 (3.9E-54)

[a] Gene numbers and functions are taken from the *Pseudomonas* genome database (59).

Table S4. Transcripts showing opposite abundance in PAO1*hfq*- and PAO1Δ*crc* when compared with PAO1.

ORF[a]	Gene	Function[a]	Fold change *hfq- vs* wt (p-value)	Fold change Δ*crc vs* wt (p-value)
PA2512	*antA*	anthranilate dioxygenase large subunit	61.5 (4.3E-131)	-15.8 (1.7E-91)
PA2514	*antB*	anthranilate dioxygenase small subunit	61.5 (5.7E-130)	-17.4 (9.4E-69)
PA2515	*antC*	anthranilate dioxygenase reductase	50.6 (9.1E-121)	-14.8 (1.8E-52)
PA0755	*opdH*	cis-aconitate porin OpdH	-12.8 (4.3E-57)	8.9 (1.6E-28)

[a] Gene numbers and functions are taken from the *Pseudomonas* genome database (59).

Table S5. Transcripts with increased abundance in strains PAO1*hfq-* and PAO1Δ*crc* when compared with PAO1

ORF[a]	Gene	Function[a]	Fold change *hfq-* vs wt (p-value)	Fold change Δ*crc vs* wt (p-value)
**Glucose metabolism**				
PA3186	*oprB*	Glucose/carbohydrate outer membrane porin OprB precursor	306.2 (2.0E-179)	164.1 (3.0E-262)
PA3187	*gltK*	probable ATP-binding component of ABC transporter	242.3 (5.6E-177)	225.3 (5.0E-280)
PA3188	*gltK*	probable permease of ABC sugar transporter	196.3 (6.1E-174)	196.2 (1.5E-258)
PA3189	*gltF*	probable permease of ABC sugar transporter	145.5 (2.7E-165)	127.1 (3.0E-238)
PA3190	*gltB*	probable binding protein component of ABC sugar transporter	222.9 (1.1E-169)	178.9 (2.6E-268)
**Gluconate/2-ketogluconate metabolism**				
PA2260	*kguE*	hypothetical protein	8.9 (3.7E-20)	6.4 (3.8E-40)
PA2261	*kguK*	probable 2-ketogluconate kinase	8.9 (2.2E-13)	5.9 (3.9E-37)
PA2262	*kguT*	probable 2-ketogluconate transporter	21.1 (6.2E-32)	12.7 (2.7E-69)
PA2263		probable 2-hydroxyacid dehydrogenase	15.1 (1.8E-27)	11.5 (1.0E-40)
PA2265	*gad*	gluconate dehydrogenase	6.3 (9.7E-35)	5.9 (1.4E-21)
**Mannitol metabolism**				
PA2338	*mtlE*	probable binding protein component of ABC maltose/mannitol transporter	12.9 (2.9E-62)	10.6 (5.0E-67)
PA2339	*mltF*	probable binding-protein-dependent maltose/mannitol transport protein	21.8 (3.7E-82)	19.3 (1.2E-114)
PA2340	*mtlG*	probable binding-protein-dependent maltose/mannitol transport protein	22.7 (4.7E-83)	20.0 (2.0E-115)
PA2341		probable ATP-binding component of ABC maltose/mannitol transporter	17.3 (1.5E-73)	16.2 (1.3E-106)
PA2342	*mtlD*	mannitol dehydrogenase	23.8 (1.1E-85)	22.3 (3.4E-124)
PA2343	*mtlY*	xylulose kinase	21.3 (7.3E-80)	20.7 (5.9E-117)
PA2344	*mtlZ*	fructokinase	16.3 (5.6E-68)	16.2 (1.8E-99)
**Ribose**				
PA1946	*rbsB*	binding protein component precursor of ABC ribose transporter	17.6 (1.9E-41)	5.5 (1.9E-29)
PA1947	*rbsA*	ribose transport protein RbsA	31.1 (5.4E-53)	9.1 (5.0E-58)

**Fructose metabolism**				
PA3560	*fruA*	phosphotransferase system, fructose-specific IIBC component	9.1 (7.8E-45)	5.8 (8.0E-41)
PA3561	*fruK*	1-phosphofructokinase	9.9 (2.2E-45)	5.7 (8.0E-36)
PA3562	*fruI*	phosphotransferase system transporter enzyme I, FruI	10.8 (1.9E-52)	5.6 (1.5E-41)
**Glycerol**				
PA3582	*glpK*	glycerol kinase	10.2 (1.2E-52)	5.0 (1.6E-41)
**Short chain aliphatic amides-Metabolism**				
PA3362	*amiS*	hypothetical protein	32.4 (7.3E-98)	14.4 (6.2E-93)
PA3363	*amiR*	aliphatic amidase regulator	36.6 (2.7E-104)	17.3 (1.2E-105)
PA3364	*amiC*	aliphatic amidase expression-regulating protein	55.4 (1.6E-123)	21.7 (3.2E-122)
PA3365	*amiB*	probable chaperone	72.9 (3.2E-136)	26.3 (1.1E-135)
PA3366	*amiE*	aliphatic amidase	27.3 (1.0E-93)	8.3 (8.8E-68)
**Polyamine metabolism**				
PA1409	*aphA*	acetylpolyamine aminohydrolase	5.1 (5.8E-19)	6.9 (1.4E-31)
PA1410	*potF1*	probable periplasmic spermidine/putrescin binding protein	8.6 (6.1E-30)	11.1 (5.6E-47)
**Other or unknown function**				
PA0529		conserved hypothetical protein	9.5 (8.1E-32)	5.7 (2.8E-26)
PA0874		hypothetical protein	37.2 (1.4E-93)	48.9 (3.5E-81)
PA1051		probable transporter	15.2 (4.4E-61)	6.6 (3.2E-39)
PA1156	*nrdA*	NrdA, catalytic component of class Ia ribonucleotide reductase	6.6 (6.3E-29)	5.0 (3.5E-42)
PA2928		hypothetical protein	6.1 (9.0E-28)	9.2 (1.3E-30)
PA2929		hypothetical protein	6.7 (2.4E-30)	21.6 (8.8E-40)
PA3570	*mmsA*	methylmalonate-semialdehyde dehydrogenase	9.4 (9.3E-40)	8.4 (4.7E-46)
PA3894		probable outer membrane protein precursor	7.4 (4.7E-37)	6.3 (3.7E-44)
PA4022	*hdhA*	hydrazone dehydrogenase, HdhA	14.8 (4.3E-31)	6.1 (5.6E-48)
PA4023		probable transport protein	86.9 (7.1E-112)	6.0 (1.0E-37)

PA4147	*acoR*	transcriptional regulator AcoR	9.7 (2.0E-16)	5.9 (1.0E-47)
PA4916		hypothetical protein	6.2 (4.2E-35)	5.7 (4.4E-48)
PA4918		Amidases related to nicotinamidase	19.1 (3.1E-81)	10.7 (1.1E-76)
PA4919	*pncB1*	nicotinate phosphoribosyltransferase	15.7 (2.4E-63)	8.3 (2.4E-70)
PA4920	*nadE*	NH3-dependent NAD synthetase	11.5 (7.7E-60)	8.8 (6.6E-74)
PA4921	*choE*	cholinesterase, ChoE	20.0 (1.5E-79)	13.1 (1.5E-30)

[a] Gene numbers and functions are taken from the *Pseudomonas* genome database (59).

Table S6. Transcripts with decreased abundance in strains PAO1*hfq-* and PAO1Δ*crc* when compared with PAO1.

**ORF**[a]	**Gene**	**Function**[a]	**Fold change *hfq- vs* wt (p-value)**	**Fold change Δ*crc vs* wt (p-value)**	**QS regulated**[b]
PA0447	*gcdH*	glutaryl-CoA dehydrogenase	-6.2 (1.1E-07)	-5.1 (1.1E-06)	*qscR*
PA0572		hypothetical protein	-12.4 (1.9E-43)	-5.2 (3.9E-25)	*las*
PA1183	*dctA*	C4-dicarboxylate transport protein	-87.0 (2.4E-145)	-51.4 (3.3E-179)	*qscR*
PA1247	*aprE*	alkaline protease secretion protein AprE	-6.8 (5.3E-38)	-11.1 (1.8E-55)	*las*
PA1248	*aprF*	alkaline protease secretion outer membrane protein AprF precursor	-6.6 (6.6E-34)	-8.5 (5.8E-40)	*las/pqs*
PA1249	*aprA*	alkaline metalloproteinase precursor	-35.0 (3.1E-110)	-27.6 (2.7E-138)	*las/pqs*
PA1656	*hsiA2*	type VI secretion protein HsiA2	-5.3 (3.2E-31)	-5.5 (7.7E-47)	*las/rhl/pqs*
PA1657	*hsiB2*	type VI secretion protein HsiB2	-8.8 (5.4E-46)	-6.7 (1.1E-51)	*las/rhl*
PA1658	*hsiC2*	type VI secretion protein HsiC2	-9.0 (5.2E-49)	-6.7 (3.2E-55)	*las/rhl*
PA1659	*hsiF2*	type VI secretion protein HsiF2	-7.6 (7.7E-24)	-5.4 (1.5E-19)	*las/rhl*
PA1665	*fha2*	type VI secretion protein Fha2	-8.8 (5.4E-23)	-6.0 (2.6E-18)	*las/rhl*
PA1666	*lip2*	type VI secretion protein Lip2	-9.4 (6.1E-43)	-6.0 (3.8E-38)	*las/rhl*
PA1871	*lasA*	LasA protease precursor	-23.7 (1.1E-16)	-7.9 (7.5E-10)	*las/rhl*
PA1899	*phzA2*	phenazine biosynthesis protein	-29.3 (3.0E-08)	-12.3 (8.6E-06)	*qscR*
PA1900	*phzB2*	phenazine biosynthesis protein	-33.9 (5.3E-13)	-14.8 (3.4E-09)	*qscR*
PA1901	*phzC2*	phenazine biosynthesis protein PhzC	-20.1 (3.7E-04)	-13.8 (1.6E-03)	*rhl/qscR*
PA2066		hypothetical protein	-11.9 (3.9E-18)	-12.2(7.4E-18)	*rhl/qscR*
PA2067		probable hydrolase	-11.4 (6.9E-20)	-9.9 (8.3E-18)	*rhl/qscR/pqs*
PA2068		probable major facilitator superfamily (MFS) transporter	-20.0 (1.2E-18)	-15.2 (3.3E-16)	*rhl/qscR*
PA2069		probable carbamoyl transferase	-41.9 (4.5E-110)	-16.9 (1.5E-80)	*rhl/qscR/pqs*
PA2300	*chiC*	chitinase	-79.3 (9.3E-36)	-26.9 (1.4E-24)	*rhl/pqs*
PA2570	*lecA*	galactophilic lectin LecA	-20.4 (1.9E-05)	-6.3 (2.7E-03)	*rhl/qscR/pqs*
PA3327		probable non-ribosomal peptide synthetase	-13.3 (1.3E-23)	-5.3 (2.3E-13)	*rhl/qscR*

PA3328		probable FAD-dependent monooxygenase	-23.0 (3.7E-86)	-6.4 (1.5E-41)	*rhl/qscR*
PA3329		hypothetical protein	-26.4 (3.8E-60)	-6.9 (3.5E-28)	*rhl/qscR*
PA3330		probable short chain dehydrogenase	-27.9 (1.1E-74)	-7.2 (2.5E-35)	*rhl/qscR*
PA3331		cytochrome P450	-28.5 (2.5E-96)	-6.9 (1.5E-57)	*rhl/qscR*
PA3332		conserved hypothetical protein	-33.9 (4.4E-99)	-7.3 (2.6E-57)	*rhl/qscR*
PA3333	*fabH2*	3-oxoacyl-[acyl-carrier-protein] synthase III	-26.7 (6.3E-93)	-6.9 (4.9E-57)	*rhl/qscR*
PA3334		probable acyl carrier protein	-25.3 (3.1E-86)	-7.4 (4.9E-57)	*rhl/qscR*
PA3335		hypothetical protein	-14.3 (1.4E-65)	-6.0 (5.3E-48)	*rhl/qscR/pqs*
PA3336		probable major facilitator superfamily (MFS) transporter	-16.0 (5.2E-37)	-6.7 (1.1E-22)	*rhl/qscR*
PA3478	*rhlB*	rhamnosyltransferase chain B	-6.6 (8.2E-13)	-8.9 (6.6E-15)	*rhl/qscR/pqs*
PA3520		hypothetical protein	-6.8 (1.0E-04)	-6.1 (2.5E-04)	*rhl/pqs*
PA3552	*arnB*	lipopolysaccharide modification protein ArnB	-11.5 (8.2E-25)	-7.9 (5.6E-20)	*qscR*
PA3553	*arnC*	lipopolysaccharide modification protein ArnC	-10.7 (6.7E-11)	-8.4 (1.7E-09)	*qscR*
PA3554	*arnA*	lipopolysaccharide modification protein ArnA	-8.7 (3.9E-13)	-8.1 (1.8E-12)	*qscR*
PA3555	*arnD*	lipopolysaccharide modification protein ArnD	-11.0 (6.3E-23)	-7.9 (1.4E-18)	*qscR*
PA3556	*arnT*	inner membrane L-Ara4N transferase	-9.7 (4.9E-48)	-6.6 (1.2E-45)	*qscR*
PA3557	*arnE*	lipopolysaccharide modification protein ArnE	-9.4 (1.3E-31)	-7.7 (1.5E-27)	*qscR*
PA3558	*arnF*	lipopolysaccharide modification protein ArnF	-10.5 (4.6E-43)	-7.8 (1.5E-43)	*qscR*
PA3559		probable nucleotide sugar dehydrogenase	-13.2 (1.1E-11)	-8.4 (3.4E-09)	*qscR*
PA4129		hypothetical protein	-42.9 (4.2E-111)	-14.6 (9.2E-97)	*las*
PA4130		probable sulfite or nitrite reductase	-42.6 (3.4E-116)	-12.2 (6.1E-92)	*las*
PA4131		probable iron-sulfur protein	-56.4 (1.1E-128)	-7.4 (1.8E-63)	*las/pqsR*
PA4132		conserved hypothetical protein	-34.2 (2.3E-106)	-7.4 (5.1E-63)	*las*
PA4133		cytochrome c oxidase subunit	-96.2 (1.4E-60)	-29.6 (5.8E-41)	*las/qscR*
PA4134		hypothetical protein	-20.6 (5.7E-30)	-16.8 (4.7E-27)	*las/qscR*
PA4139		hypothetical protein	-14.4 (2.3E-09)	-8.5 (5.9E-07)	*las*

PA4141		hypothetical protein	-17.0 (5.7E-77)	-5.4 (1.2E-47)	*rhl/qscR/pqs*
PA4217	*phzS*	flavin-containing monooxygenase	-6.2 (2.5E-25)	-8.5 (6.9E-31)	*rhl/qscR/pqs*
PA4773		hypothetical protein	-13.8 (1.8E-03)	-10.1 (4.8E-03)	*qscR*
PA4774		hypothetical protein	-18.6 (2.9E-05)	-12.7 (1.6E-04)	*qscR*
PA4775		hypothetical protein	-6.7 (1.3E-05)	-6.3 (2.1E-05)	*qscR*
PA5383		conserved hypothetical protein	-22.0 (1.2E-03)	-21.0 (1.8E-03)	*qscR*

[a] Gene numbers and functions are taken from the *Pseudomonas* genome database (59).

[b] Genes regulated by the *las*, *rhl* or *pqs* quorum-sensing systems and/or by the orphan quorum sensing regulator QscR according to Schuster *et al*. (68), Lequette *et al*. (69), Déziel *et al*. (70), Bredenbruch *et al*. (71), and Wang *et al*. (72), respectively.

Table S7. Protein-Protein cross-links observed in the Hfq/Crc/*amiE*$_{6ARN}$ complex

cross-linked proteins	cross-linked residues	Peptide sequence 1[a]	Peptide sequence 2[a]	frequency of occurrence	Intramolecular distance [Å]
**Trypsin digestion**					
Crc-Crc	K101-K135	YLQADFD**K**VSIATLLLPSGQSGDESLNQK	FMDDFTHYLS**K**QR	56	25.24
Crc-Crc	K124-K236	F**K**FMDDFTHYLSK	NA**K**LPR	11	38.27
Crc-Crc	K135-K236	FMDDFTHYLS**K**QR	NA**K**LPR	7	36.37
Crc-Crc	K135-K236	FKFMDDFTHYLS**K**QR	NA**K**LPR	1	36.37
Crc-Crc	K77-K155	LQP**K**AVISGLGFETADR	EYIYCGSLYVAHQ**K**MDVK	7	44.25
Crc-Crc	K77-K155	LQP**K**AVISGLGFETADR	REYIYCGSLYVAHQ**K**MDVK	1	44.25
Crc-Crc	K77-K135	LQP**K**AVISGLGFETADR	FMDDFTHYLS**K**QR	3	21.90
Crc-Crc	K124-K155	F**K**FMDDFTHYLSK	EYIYCGSLYVAHQ**K**MDVK	3	31.05
Crc-Crc	K159-K236	MDV**K**NWR	NA**K**LPR	3	17.77
Crc-Crc	K135-K155	FMDDFTHYLS**K**QR	EYIYCGSLYVAHQ**K**MDVK	2	34.79
Crc-Crc	K155-K236	REYIYCGSLYVAHQ**K**MDVK	NA**K**LPR	2	25.86
Crc-Crc	K101-K236	YLQADFD**K**VSIATLLLPSGQSGDESLNQK	NA**K**LPR	1	28.71
Crc-Crc	K135-K135	FMDDFTHYLS**K**QR	FMDDFTHYLS**K**QR	1	0
Crc-Crc	M1-K155	**M**RIISVNVNGIQAAAER	REYIYCGSLYVAHQ**K**MDVK	1	33.41
Crc-Crc	K155-K155	REYIYCGSLYVAHQ**K**MDVK	EYIYCGSLYVAHQ**K**MDVK	1	0
Crc-Crc	K155-K159	EYIYCGSLYVAHQ**K**MDVK	MDV**K**NWRECQQMPGFLAPER	1	14.77
Crc-Crc	K236-K236	FVRNA**K**LPR	NA**K**LPRQPR	1	0
Hfq-Crc	K3-K236	S**K**GHSLQDPYLNTLR	NA**K**LPR	1	
**Trapsin and Lys-C digestion**					
Crc-Crc	K77-K101	LQP**K**AVISGLGFETADR	YLQADFD**K**VSIATLLLPSGQSGDESLNQK	129	10.85
Crc-Crc	K77-K101	LQP**K**AVISGLGFETADRYGR	YLQADFD**K**VSIATLLLPSGQSGDESLNQK	3	10.85
Crc-Crc	K101-K236	YLQADFD**K**VSIATLLLPSGQSGDESLNQK	NA**K**LPR	44	28.71
Crc-Crc	K122-K236	VSIATLLLPSGQSGDESLNQ**K**FK	NA**K**LPR	39	28.24
Crc-Crc	K77-K236	LQP**K**AVISGLGFETADR	NA**K**LPR	38	31.28
Crc-Crc	K77-K236	LQP**K**AVISGLGFETADRYGR	NA**K**LPR	2	31.28
Crc-Crc	K236-K236	NA**K**LPR	NA**K**LPR	13	0
Crc-Crc	K236-K236	NA**K**LPRQPR	NA**K**LPR	1	0
Crc-Crc	K77-K122	LQP**K**AVISGLGFETADR	VSIATLLLPSGQSGDESLNQ**K**FK	13	33.08
Crc-Crc	K101-K135	YLQADFD**K**VSIATLLLPSGQSGDESLNQK	FMDDFTHYLS**K**QR	8	25.24
Crc-Crc	K101-K135	YLQADFD**K**VSIATLLLPSGQSGDESLNQK	FKFMDDFTHYLS**K**QR	1	25.24
Crc-Crc	K77-K77	LQP**K**AVISGLGFETADR	LQP**K**AVISGLGFETADR	7	0
Crc-Crc	K77-K135	LQP**K**AVISGLGFETADR	FMDDFTHYLS**K**QR	4	21.90
Crc-Crc	K122-K135	VSIATLLLPSGQSGDESLNQ**K**FK	FMDDFTHYLS**K**QR	3	22.59
Crc-Crc	K124-K236	F**K**FMDDFTHYLSK	NA**K**LPR	3	38.27
Crc-Crc	K135-K236	FKFMDDFTHYLS**K**QRR	NA**K**LPR	1	36.37
Crc-Crc	K101-K122	YLQADFD**K**VSIATLLLPSGQSGDESLNQK	VSIATLLLPSGQSGDESLNQ**K**FK	1	36.05
Crc-Crc	K159-K236	MDV**K**NWR	NA**K**LPR	1	17.77
Hfq-Hfq	K3-K17	S**K**GHSLQDPYLNTLR	GHSLQDPYLNTLRKER	5	12.72
Hfq-Crc	K3-K124	S**K**GHSLQDPYLNTLR	F**K**FMDDFTHYLSK	1	
Hfq-Crc	M1-K122	**M**SKGHSLQDPYLNTLR	VSIATLLLPSGQSGDESLNQ**K**FK	1	
Hfq-Crc	K17-K77	KERVPVSIYLVNGI**K**	LQP**K**AVISGLGFETADR	1	

[a] The BS3 cross-linked residues are shown in bold. Possible intramolecular cross-links are highlighted in blue. Hfq-Crc crosslinks are highlighted in green.

Table S8. Protein-Protein cross-links in the absence of RNA.

cross-linked proteins	cross-linked residues	Peptide sequence 1[a]	Peptide sequence 2[a]	frequency of occurrence	Intramolecular distance [Å]
Crc-Crc	K135-K135	FMDDFTHYLS**K**QR	FMDDFTHYLS**K**QR	11	0
Crc-Crc	K77-K155	LQP**K**AVISGLGFETADR	EYIYCGSLYVAHQ**K**MDVK	9	44.25
Crc-Crc	K77-K135	LQP**K**AVISGLGFETADR	FMDDFTHYLS**K**QR	7	21.90
Crc-Crc	K135-K155	FMDDFTHYLS**K**QR	EYIYCGSLYVAHQ**K**MDVK	4	34.79
Crc-Crc	K77-K236	FMDDFTHYLS**K**QR	NA**K**LPR	1	31.28
Crc-Crc	K101-K236	YLQADFD**K**VSIATLLLPSGQSGDESLNQK	NA**K**LPRQPR	1	28.71
Crc-Crc	K155-K236	REYIYCGSLYVAHQ**K**MDVK	NA**K**LPR	1	25.86
Crc-Crc	K124-K236	F**K**FMDDFTHYLSKQRR	NA**K**LPR	1	38.27

[a] The BS3 cross-linked residues are shown in bold. Digestion of Hfq and Crc was performed with trypsin.

Table S9. Substrate utilization by PAO1 *hfq*, *crc* and *crcZ* mutants

	Respiratory activity[b]			
**Substrate[a]**	**PAO1**	**PAO1*hfq-***	**PAO1Δ*crc***	**PAO1Δ*crcZ***
**TCA cycle intermediates**				
Cis-Aconitic acid	++	++	++	++
Citric acid	++	++	++	++
α-Keto glutaric acid	++	++	++	++
Malonic acid	++	++	++	++
Succinic acid	++	++	++	+
**Amino acids**				
D-Alanine	++	++	++	–
L-Alanine	++	++	++	–
L-Aspartic acid	++	++	++	+
L-Asparagine	++	++	++	+
L-Glutamic acid	++	++	++	++
L-Histidine	++	++	++	+
L-Leucine	+	++	+	–
L-Ornithine	++	++	++	–
L-Proline	++	–	++	+
**Sugar and Sugar alcohols**				
D-Fructose	++	++	++	–
D-Gluconic acid	++	++	++	–
D-Glucose	++	++	++	+
Glycerol	++	++	++	–
D-mannitol	++	++	++	–
**Other C sources**				
γ-Aminobutyric acid	++	++	++	+
Bromosuccinic acid	++	++	++	–
β-Hydroxybutyric acid	++	++	++	–
p-Hydroxyphenylacetic acid	++	++	++	+
Itaconic acid	++	++	++	–
D,L-Lactic acid	++	++	++	++
Propionic acid	++	++	++	–
Quinic acid	++	++	++	++
Sebacic Acid	++	++	++	–
Succanimic acid	++	++	++	–

[a] The respiratory activity of different C-sources was tested by GN2 microplates (Biolog) (for more details see Text S1).

[b] ++, respiratory activity within 24 h; +, respiratory activity within 48 h; –, no respiratory activity within 48 h.

Table S10. PAO1Δ*crcZ* revertants isolated in the presence of different C/N sources.

Name	Growth[a]	base[b]	Alterations in Crc or Hfq[c]
**Mutations in *hfq***			
**Point mutations causing amino acid substitutions**			
sup 34	L-Histidine	C_{+190} to T	Hfq_{P64S}
**Mutations in *crc***			
**Point mutations causing amino acid substitutions**			
sup 29	L-Histidine	T_{+446} to G	Crc_{L149R}
sup 2b7	L-Histidine	T_{+305} to A	Crc_{V102E}
sup A	L-Histidine	C_{+227} to T	Crc_{P76L}
sup E	Mannitol	C_{+674} to T	Crc_{T225I}
sup G	Alanine	C_{+421} to C	Crc_{R141C}
sup M	Acetamide	C_{+421} to A	Crc_{R141S}
**Point mutations resulting in premature stop codons**			
sup 5	Mannitol	C_{+282} to A	$Crc_{Y94Ochre}$
sup 15	Acetamide	C_{+282} to A	$Crc_{Y94Ochre}$
sup 28	L-Histidine	G_{+608} to A	$Crc_{W203Amber}$
sup 41	Alanine	C_{+282} to A	$Crc_{Y94Ochre}$
sup F	Mannitol	C_{+76} to T	$Crc_{Q26Amber}$
sup H	Alanine	G_{+631} to T	$Crc_{E211Amber}$
sup I	Alanine	C_{+96} to A	$Crc_{C32Opal}$
sup J	L-Histidine	G_{+631} to T	$Crc_{E211Amber}$
sup K	L-Histidine	G_{+631} to T	$Crc_{E211Amber}$
sup L	Acetamide	C_{+429} to A	$Crc_{Y143Ochre}$
**Deletions**			
sup 42	Alanine	Deletion of 273 bp from -2 to +271	Crc, deletion of the first 91aa

sup 1b5	L-Histidine	Deletion of 3 bp from +562 to +564	$Crc_{\Delta A188}$
sup 3a3	L-Histidine	Deletion of 6 bp from +74 to +79	$Crc_{\Delta A25, \Delta Q26, N27D}$
sup O	Mannitol	Deletion of 90 bp from +556 to +645	$Crc_{\Delta G186\ to\ L215}$
**Mutations causing an extension of the open reading frame**			
Sup 2a4	L-Histidine	A_{+780} to G	$Crc_{Opal260W;}$ addition of 21aa
**Frameshift mutations**			
sup D	Acetamide	Deletion of C_{+750}	Frameshift after aa L_{249} in Crc
sup N	Acetamide	Insertion of C_{+678}	Frameshift after aa P_{226} in Crc
sup P	Mannitol	Deletion of T_{+33}	Frameshift after aa G_{10} in Crc
**No mutation found in *hfq* or *crc***			
sup 4a1	L-Histidine		
sup B	L-Histidine		

[a] C/N source provided.

[b] The nucleotides are given with respect to A (+1) of the ATG start codon of the *hfq* and *crc* mRNAs, respectively.

[c] The numbers correspond to amino acid (aa) positions in Hfq and Crc, respectively.

Table S11. The complementation of PAO1Δ*crcZ* suppressor mutants with plasmid encoded *crc* or *hfq* restores translational repression of *amiE::lacZ* translation.

**Strain**	**β-galactosidase activity (Miller Units)**[a]
PAO1(pME9655,pME4510)	633 ± 262
PAO1Δ*crcZ*(pME9655,pME4510)	43 ± 37
PAO1Δ*hfq*(pME9655,pME4510)	7266 ± 164
PAO1Δ*hfq*(pME9655,pME4510hfq_{Flag})	452 ± 11
PAO1Δ*crc*(pME9655,pME4510)	4949 ± 463
PAO1Δ*crc*(pME9655,pME4510crc_{Flag})	196 ± 4
PAO1Δ$crcZ_{\mathrm{sup34}}$(pME9655,pME4510)	3076 ± 562
PAO1Δ$crcZ_{\mathrm{sup34}}$(pME9655,pME4510hfq_{Flag})	658 ± 180
PAO1Δ$crcZ_{\mathrm{sup29}}$(pME9655,pME4510)	3014 ± 241
PAO1Δcrc_{sup29}(pME9655,pME4510crc_{Flag})	6 ± 1
PAO1Δ$crcZ_{\mathrm{sup2b7}}$(pME9655,pME4510)	1712 ± 247
PAO1Δcrc_{sup2b7}(pME9655,pME4510crc_{Flag})	1 ± 0
PAO1Δ$crcZ_{\mathrm{supA}}$(pME9655,pME4510)	1943 ± 68
PAO1Δcrc_{supA}(pME9655,pME4510crc_{Flag})	24 ± 11
PAO1Δ$crcZ_{\mathrm{supE}}$(pME9655,pME4510)	3662 ± 101
PAO1Δcrc_{supE}(pME9655,pME4510crc_{Flag})	27 ± 20
PAO1Δ$crcZ_{\mathrm{supG}}$(pME9655,pME4510)	3194 ± 754
PAO1Δcrc_{supG}(pME9655,pME4510crc_{Flag})	10 ± 4

[a] The strains were grown to an OD_{600} of 2.0 in BSM medium supplemented with 40 mM succinate and 40 mM acetamide (transcriptional induction of *amiE::lacZ* reporter gene). The β-galactosidase values conferred by the translational *amiE::lacZ* fusion encoded by plasmid pME9655 in the respective strains are indicated. In addition to plasmid pME9655, the strains were also transformed with either the empty vector pME4510 or plasmids pME4510crc_{Flag} and pME4510hfq_{Flag}, respectively, to complement the *crc* and *hfq* mutations of the respective revertants. The results represent data from two independent experiments and are shown as mean ± standard deviation.
